# Machine Learning Prediction of Multidrug Resistance in Swine-Derived *Campylobacter* spp. Using United States Antimicrobial Resistance Surveillance Data (2013–2023)

**DOI:** 10.3390/vetsci12100937

**Published:** 2025-09-26

**Authors:** Hamid Reza Sodagari, Maryam Ghasemi, Csaba Varga, Ihab Habib

**Affiliations:** 1 Independent Researcher, Perth 6107, Australia; 2 Department of Quality Assurance and Data Analytics, Jouya Behnood Company, Tehran, Iran; ghasemi.maryam@jouyabehnood.com; 3 Department of Pathobiology, College of Veterinary Medicine, University of Illinois Urbana-Champaign, Urbana, IL 61802, USA; cvarga@illinois.edu; 4 Carl R. Woese Institute for Genomic Biology, University of Illinois Urbana-Champaign, Urbana, IL 61802, USA; 5 Veterinary Public Health Research Laboratory, Department of Veterinary Medicine, College of Agriculture and Veterinary Medicine, United Arab Emirates University, Al Ain P.O. Box 15551, United Arab Emirates

**Keywords:** machine learning, multidrug resistance, *Campylobacter*, swine, predictive modeling, classification algorithms, United States

## Abstract

*Campylobacter* spp. are leading causes of bacterial gastroenteritis globally. Swine are recognized as an important reservoir for this pathogen. The emergence of antimicrobial resistance (AMR) and multidrug resistance (MDR) in *Campylobacter* is a global health concern. Traditional methods for detecting AMR and MDR, such as phenotypic testing or whole-genome sequencing, are resource-intensive and time-consuming. In the present study, we developed and validated a supervised machine learning model to predict MDR status in *Campylobacter* isolates from swine, using publicly available phenotypic AMR data collected by NARMS from 2013 to 2023. Resistance profiles for seven antimicrobials were used as predictors, and MDR was defined as resistance to at least one agent in three or more antimicrobial classes. The model was trained on 2013–2019 isolates and externally validated using isolates from 2020, 2021, and 2023. Random Forest showed the highest performance (accuracy = 99.87%, Kappa = 0.9962) among five evaluated algorithms, which achieved high balanced accuracy, sensitivity, and specificity in both training and external validation. Our feature importance analysis identified erythromycin, azithromycin, and clindamycin as the most influential predictors of MDR among *Campylobacter* isolates from swine. Our temporally validated, interpretable model provides a robust, cost-effective tool for predicting MDR in *Campylobacter* spp. and supports surveillance and early detection in food animal production systems.

## Simple Summary

*Campylobacter* spp. are among the most important causes of bacterial gastroenteritis around the world. In addition to poultry, pigs are also considered an important source of this pathogen. Antimicrobial resistance (AMR) in *Campylobacter* is a serious public health concern. A supervised machine learning model was developed and validated in this study to predict MDR status in *Campylobacter* isolates from swine, using publicly available phenotypic AMR data collected by NARMS from 2013 to 2023. Among five evaluated machine learning algorithms, Random Forest showed the highest performance (accuracy = 99.87%, Kappa = 0.9962), achieving high balanced accuracy, sensitivity, and specificity in both training and external validation. The feature importance analysis found that erythromycin, azithromycin, and clindamycin were the most influential predictors of MDR among *Campylobacter* isolates from swine. Our temporally validated, interpretable model offers a robust and cost-effective approach for predicting MDR in *Campylobacter* spp., facilitating surveillance and early detection in food animal production systems.

## 1. Introduction

*Campylobacter* spp. has been recognized as one of the leading causes of bacterial gastroenteritis globally [1]. Campylobacteriosis in humans is mostly caused by *Campylobacter jejuni* and *Campylobacter coli*, which are commonly found in the gastrointestinal tract of poultry, livestock, and other animals [2]. Infections with this pathogen are often associated with the consumption of contaminated food products, especially undercooked or raw poultry, unpasteurized milk, and untreated water [3,4,5]. In addition to poultry, swine have also been recognized as a reservoir of *Campylobacter* spp. [6]. While most *Campylobacter* spp. infections are self-limiting, in rare cases, they can cause reactive arthritis, Guillain–Barré syndrome, or post-infectious irritable bowel syndrome [7,8]. In severe forms of *Campylobacter* infections or for patients with an increased risk of developing complications, macrolides and fluoroquinolones may be prescribed as the drugs of choice for treatment [9].

The emergence of antimicrobial resistance (AMR) and multidrug resistance (MDR) is a global health concern, influencing the effectiveness of treatment of infections in both human and veterinary medicine [10]. Several previous studies have consistently documented rising MDR trends in animal-derived *Campylobacter* spp. isolates, including poultry [11,12] and swine [13]. The emergence of resistance to macrolides (azithromycin, erythromycin) and fluoroquinolones (ciprofloxacin) is concerning, as they are commonly prescribed to treat severe *Campylobacter* spp. infections [14].

Detection of AMR and MDR relies on isolating bacterial pathogens from cases and susceptibility testing of isolates to a panel of antimicrobials or conducting whole-genome sequencing (WGS), which are resource-intensive and time-consuming [15]. Consequently, there is growing interest in computational approaches that enhance the predictive capabilities of existing surveillance data [16]. Machine learning has emerged as a powerful approach for pattern recognition and classification in complex biological datasets [17]. Several recent investigations have indicated the application of machine learning models in predicting AMR in human pathogens using phenotypic, genomic, or clinical metadata [18,19,20,21]. Various supervised and unsupervised machine learning models have demonstrated AMR-predictive performance across different pathogens. For instance, machine learning models have predicted carbapenem-resistance in Gram-negative bacteria among ICU patients with accuracies of 72–84% [22]. In another study, an open-source machine learning algorithm (XGBoost, Version 3.0.5) was applied to predict antibiotic resistance in three Gram-negative bacterial species (*E. coli*, *Klebsiella pneumoniae,* and *Pseudomonas aeruginosa*) isolated from patients’ blood and urine [23]. In a separate study, a Random Forest model has also been used to accurately predict AMR in *Pseudomonas aeruginosa* [24]. Focusing on *Campylobacter* spp., a machine learning model (Support Vector Machine) on protein sequences successfully predicted resistance genes in Gram-negative bacteria, including *Campylobacter* spp., with 90% accuracy [25]. In another investigation, a combination of Matrix-Assisted Laser Desorption/Ionization-Time of Flight (MALDI-TOF) mass spectrometry and machine learning showed high performance in detecting susceptible, as well as ciprofloxacin- and tetracycline-resistant *Campylobacter* spp. isolates from clinical and environmental sources [26].

Although machine learning-based AMR research is rapidly growing, few studies have investigated machine learning-based MDR prediction in pathogens derived from livestock using phenotypic data. Therefore, this study utilizes publicly available data on AMR in *Campylobacter* spp. isolated from cecal samples of swine at slaughter collected by the National Antimicrobial Resistance Monitoring System of Enteric Bacteria (NARMS) between 2013 and 2023 to: (i) identify the machine learning algorithm with the highest MDR predictive accuracy, (ii) quantify the contribution of individual AMR traits to MDR classification, and (iii) evaluate temporal robustness of the predictions by testing model performance using isolates not included in the model training, which were collected during the final three years of the study period. The value of this work could be extended to additional livestock sectors and might have global application.

## 2. Materials and Methods

### 2.1. Study Design

**Figure 1:**
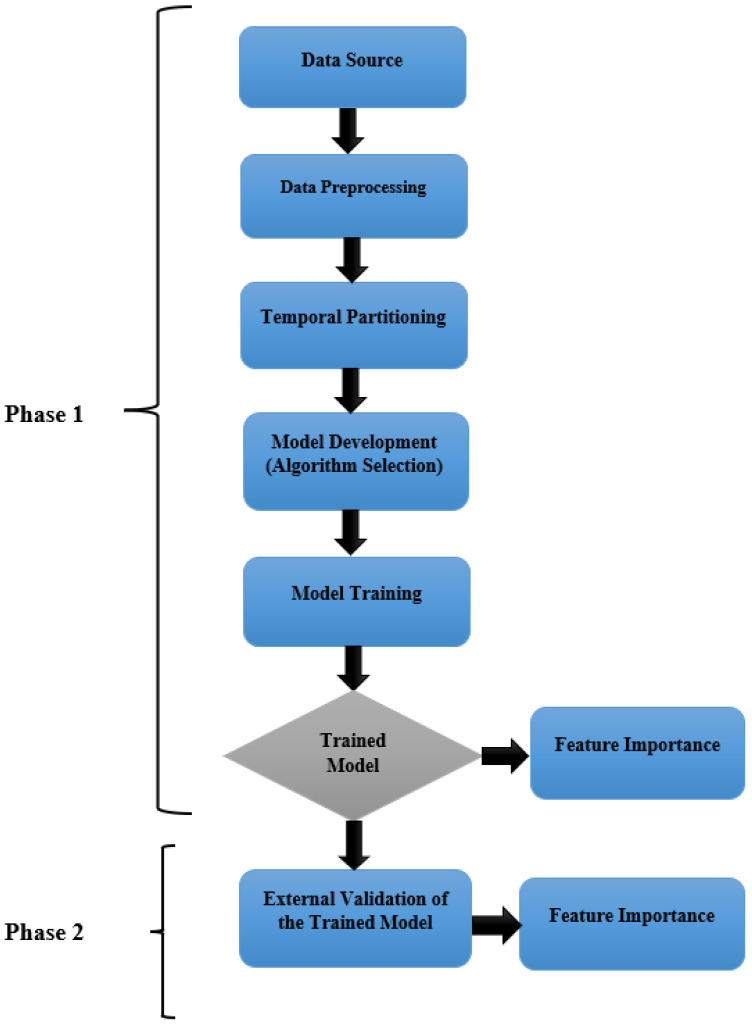
Workflow of the machine learning pipeline to develop and validate a predictive model for multidrug resistance (MDR) in swine-derived *Campylobacter* isolates.

In this study, we developed and validated a supervised machine learning model to predict MDR status in the US swine-derived *Campylobacter* spp. isolates. The investigation used publicly available AMR monitoring data collected by NARMS [27]. This dataset includes AMR data of *Campylobacter* spp. isolates from the cecal content of swine at Food Safety and Inspection Service—U.S. Department of Agriculture (USDA)-regulated slaughter establishments across the US from 2013 to 2023 [27]. Antimicrobial susceptibility results (resistant or susceptible) of each isolate were recorded for seven antimicrobials included in this study: azithromycin (AZI), clindamycin (CLI), erythromycin (ERY), ciprofloxacin (CIP), nalidixic acid (NAL), tetracycline (TET), and gentamicin (GEN). Resistance to each antimicrobial was recorded as binary (1 = resistant, 0 = susceptible). The outcome variable was MDR, defined as resistance to at least one agent in three or more antimicrobial classes [28], and was encoded as a binary classification (YES, NO). Figure 1 shows the workflow of our machine learning pipeline, including data preprocessing, algorithm selection, model training, and validation steps.

Our dataset was temporally partitioned into two phases (Phase 1 and Phase 2) to simulate a real-world application and evaluate the model’s generalizability over time.

Phase 1 included isolates collected from 2013 to 2019, which were used for model development and internal validation. Phase 2 consisted of isolates from the last three years of the study period (2020, 2021, 2023) and served as an external test dataset for independent validation (data for the year 2022 was not available). The “Year” variable was only used for temporal stratification and excluded from model training.

### 2.2. Phase 1

#### 2.2.1. Data Preprocessing and Temporal Partitioning

Before model training, the dataset was cleaned by removing non-informative identifiers. The MDR labels were converted to a factor variable. While scaling and centering are typically required for distance-based algorithms such as Support Vector Machines (SVM) and k-Nearest Neighbors (KNN) [29], these steps were not applied, as all predictors in our dataset were binary [29]. All analysis was performed using R software (Version 4.5.0, 11-04-2025) [30], within the RStudio platform (2024.09.0 Build 375 © 2009–2024 Posit Software, PBC, Boston, MA, USA).

#### 2.2.2. Algorithm Selection and Internal Validation

Five supervised classification machine learning algorithms, including Support Vector Machine (SVM), Random Forest, Decision Tree, Naive Bayes, and k-Nearest Neighbors (KNN), which have been extensively used in previous studies [31,32,33,34], were evaluated for their ability to predict MDR status in *Campylobacter* spp. isolates from swine based on resistance patterns of predictor variables (seven above-mentioned antimicrobials). All models were trained using 5-fold cross-validation to ensure robust performance estimates. Cross-validation was performed using the “caret R-package”, and model accuracy and Cohen’s Kappa were used for comparative evaluation. Resampling summaries were generated to evaluate the distribution of performance metrics across folds. Hyperparameter tuning for each algorithm was performed automatically using the default grid search provided by the caret package during 5-fold Cross-Validation.

#### 2.2.3. Model Development and Validation Using the Selected Machine Learning Algorithm

Among the evaluated supervised machine learning algorithms, the one with the highest cross-validated accuracy and Kappa scores was selected to develop the final predictive model. The dataset (2013–2019) was initially partitioned into training (80%) and testing (20%) subsets using stratified sampling to preserve the distribution of MDR classes. The selected algorithm was then trained and internally tested on the training subset (2013–2019), and the final trained model was saved for external validation.

A five-fold cross-validation was conducted on the entire dataset to assess model robustness and performance. In each fold, the data were split into training (80%) and validation (20%) subsets. The model was trained on the training portion and evaluated on the validation subset. During training, feature standardization techniques (centering and scaling) were applied to improve model performance.

Predictions from all folds were aggregated to produce out-of-fold predictions across the entire dataset. These predictions were compared to the true MDR labels to generate a single overall confusion matrix. Performance metrics, including accuracy, sensitivity, specificity, positive predictive value (PPV), negative predictive value (NPV), and Cohen’s kappa, were computed to assess the developed model’s performance. The confusion matrix was then visualized using the ggplot2 package, showing assessment of misclassification and class imbalance.

#### 2.2.4. Feature Importance Analysis

To identify the most influential predictors of MDR, feature importance scores were extracted from the trained Random Forest model using the RandomForest R-package. These scores represent the relative contribution of each antibiotic resistance feature (seven antimicrobials included in this study) to the prediction of MDR status. Features were ranked by their mean decrease in Gini impurity and then visualized using the ggplot2 package to identify the most influential predictors.

### 2.3. Phase 2

#### External Validation of the Trained Model

To evaluate potential overfitting and assess temporal generalizability, an external validation was performed by splitting the dataset chronologically. Isolates from 2013 to 2019 were already used to train the model (Phase 1), while isolates from the last three years of the study period (2020, 2021, and 2023), which have not been used to train the model, were reserved for external validation (Phase 2). The ‘Year’ column was excluded from predictor variables (AMR status of the antimicrobials that were included) to prevent data leakage. The trained Random Forest model was applied to the test set, and predictive performance metrics and a confusion matrix were generated as described in phase 1.

Feature importance analysis was also performed using the same method described in phase 1 to identify the most influential predictors based on their feature importance score, contributing to MDR classification during external validation. The results were compared to those from phase 1 (model training step) to identify any shift in predictive patterns.

## 3. Results

### 3.1. Performance Evaluation of Classification Machine Learning Algorithms for MDR Prediction in Swine-Derived Campylobacter

**Figure 2:**
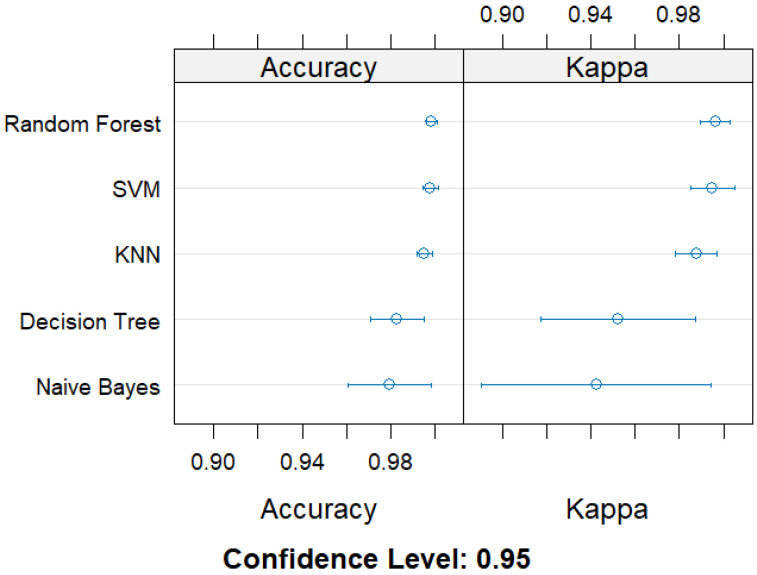
Cross-validation performance metrics (Accuracy and Cohen’s Kappa) of five supervised machine learning algorithms for predicting multidrug resistance in *Campylobacter* spp. isolates from swine.

We initially evaluated five classification machine learning algorithms: SVM, Decision Tree, Random Forest, Naive Bayes, and KNN using 5-fold cross-validation. Among the evaluated algorithms, the Random Forest algorithm exhibited the highest performance on our dataset (Accuracy = 99.87%, Kappa = 0.9962), followed by SVM (Accuracy = 99.82%, Kappa = 0.9950) and KNN (Accuracy = 99.55%, Kappa = 0.9876) (Figure 2, Supplementary Table S1). Our results also showed that the lowest-performing algorithms were the Decision Tree (Accuracy = 98.31%, Kappa = 0.9520) and Naive Bayes (Accuracy = 97.96%, Kappa = 0.9423) (Figure 2, Supplementary Table S1). Based on our evaluation, the Random Forest algorithm was selected for developing the model (using the Random Forest R-package) and further analysis due to its high accuracy and robustness across folds.

### 3.2. Development and Evaluation of a Random Forest Model to Predict Multidrug Resistance in Campylobacter from Swine

**Figure 3:**
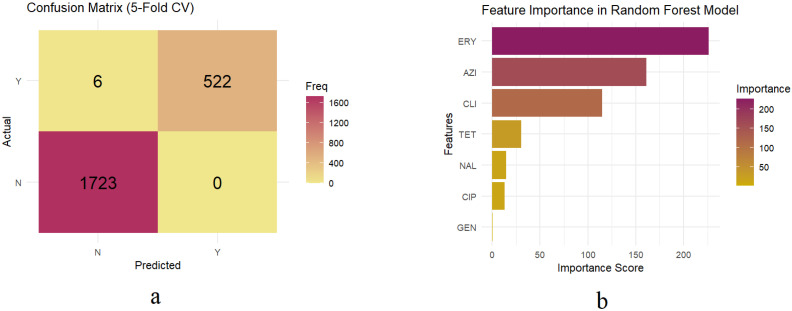
(**a**) Confusion matrix showing predicted versus actual multidrug resistance classification results of the trained Random Forest model using 5-fold cross-validation. The matrix displays the number of correctly and incorrectly classified MDR (Y) and non-MDR (N) isolates. (**b**) Feature importance scores of predictors from the trained Random Forest model predicting MDR in swine-derived *Campylobacter* isolates. ERY: erythromycin, AZI: azithromycin, CLI: clindamycin, TET: tetracycline, NAL: nalidixic acid, CIP: ciprofloxacin, GEN: gentamicin.

The developed Random Forest model was then evaluated using a 5-fold cross-validation strategy. The model showed excellent classification performance, with a balanced accuracy of 99.43% and a Kappa score of 0.9925 (Table 1). The model also achieved high sensitivity (98.86%), specificity (100%), PPV (100%), and NPV (99.65%) (Table 1). The confusion matrix represented that our trained model had high precision with only 6 misclassified isolates out of 2551, all of which were MDR isolates incorrectly predicted as non-MDR (Figure 3a, Supplementary Table S2).

### 3.3. Important Features Predicting MDR in the Trained Random Forest Model

Feature importance score for the predictors (resistance to a specific antimicrobial) obtained from the trained Random Forest model demonstrated the relative contribution of each AMR feature in predicting MDR status (Figure 3b). Among the evaluated predictors, the macrolide antimicrobial class, including erythromycin (importance score = 226.40) and azithromycin (importance score = 161.10), along with the lincosamide class, including clindamycin with a score of 115.14, were the most influential predictors of MDR in *Campylobacter* spp. isolates (Figure 3b). Other predictors, including tetracycline, nalidixic acid, ciprofloxacin, and gentamicin, indicated lower importance scores of 30.13, 14.78, 12.70, and 0.45, respectively (Figure 3b).

### 3.4. External Validation of the Trained Random Forest Model (Phase 2)

**Figure 4:**
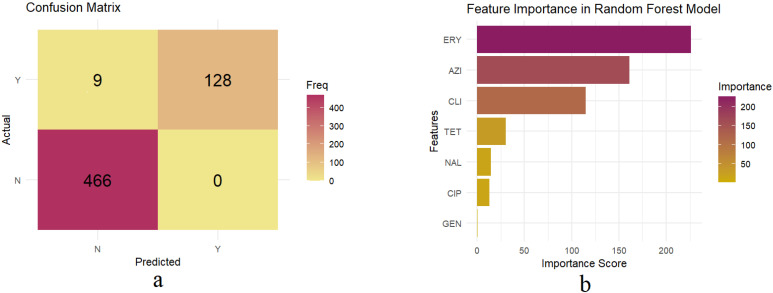
(**a**) Confusion matrix showing predicted versus actual MDR classification results from the external validation of the trained Random Forest model. The matrix displays the number of correctly and incorrectly classified MDR (Y) and non-MDR (N) isolates. (**b**) Feature importance scores of predictors from the external validation dataset, evaluating the trained Random Forest model predicting MDR in swine-derived *Campylobacter* spp. isolates. ERY: erythromycin, AZI: azithromycin, CLI: clindamycin, TET: tetracycline, NAL: nalidixic acid, CIP: ciprofloxacin, GEN: gentamicin.

To evaluate model generalizability and identify potential overfitting, the trained Random Forest model from Phase 1 was applied to predict MDR outcomes on a new test dataset (n = 603 isolates) from the last three years of the study period (2020, 2021, 2023), which was not used during model training. Our external validation showed that the model achieved a high balanced accuracy of 96.72% and a Kappa score of 0.9565 (Table 2). High sensitivity (93.43%) and maximum specificity (100%) (Table 2), further reinforcing the high validity and predictive power of our trained model on temporally independent data. Despite a slight performance decline compared to the previous phase (phase 1), our trained model indicated high precision with 9 misclassified isolates out of 603, which were incorrectly predicted as non-MDR isolates (Figure 4a, Supplementary Table S3). Table 2 and Figure 4a show external validation results, including performance statistics and a confusion matrix of our Random Forest model.

### 3.5. Important Features Predicting MDR in the External Validation Phase of the Trained Random Forest Model

Feature importance scores of the predictors for the external validation dataset were consistent with phase 1. Erythromycin (importance score = 226.40), azithromycin (importance score = 161.10), and clindamycin (importance score = 1115.14) were the most influential predictors of MDR in *Campylobacter* spp. isolates (Figure 4b). Tetracycline, nalidixic acid, ciprofloxacin, and gentamicin were also other features indicating lower importance scores of 30.13, 14.78, 12.70, and 0.45, respectively (Figure 4b).

## 4. Discussion

In this study, using phenotypic AMR data collected by NARMS, we developed and validated a supervised machine learning model to predict MDR status in swine-derived *Campylobacter* spp. isolates, by including predictors representing the isolates’ antimicrobial resistance status to seven antimicrobials (erythromycin, azithromycin, clindamycin, tetracycline, nalidixic acid, ciprofloxacin, and gentamicin). The model identified that resistance to erythromycin, azithromycin, and clindamycin was the most influential predictor of MDR in *Campylobacter* spp. isolates, as this was the highest importance score compared to the other predictors.

Among the five classification machine learning algorithms evaluated in the present study, the Random Forest algorithm represented the highest performance (99.87% accuracy) and was selected for model development. Our trained Random Forest model demonstrated strong performance, achieving a balanced accuracy of 99.43% on the training dataset from 2013 to 2019 (Phase 1) and maintaining a high accuracy of 96.72% when tested on the external dataset from 2020, 2021, and 2023 (Phase 2). These findings suggest that supervised classification algorithms may provide a robust, data-driven approach to predict MDR status in *Campylobacter* spp. using phenotypic AMR surveillance data. Similar trends have been observed in previous studies employing machine learning for AMR prediction in *Campylobacter*. Chowdhury et al. (2019) applied SVM model to predict antimicrobial resistance genes in Gram-negative bacteria, including *Campylobacter*, and reported an accuracy above 90% [25]. In another investigation, gradient boosting (XGBoost) regression was used to predict AMR in *C. jejuni* and other pathogens, achieving accurate metrics comparable to our findings [35]. Several previous machine learning investigations primarily focused on other pathogens like *M. tuberculosis*, *E. coli*, *S. aureus*, and *Klebsiella*, and only examined resistance to a single class of antimicrobial rather than predicting MDR [36,37,38,39].

The present study indicated high sensitivity (98.86%) and maximum specificity (100%) in predicting MDR status among swine-derived *Campylobacter*. Misclassifying a resistant isolate as susceptible can result in ineffective treatment, potentially influencing infection management in patients [26]. Therefore, classifiers that inform antimicrobial therapy decisions must prioritize high sensitivity [40]. The high sensitivity achieved in both training the model and its external validation step in this study highlights the robustness of the MDR predictive approach.

Many existing AMR and MDR prediction tools rely on genomic data. Although these approaches are powerful, they often require specialized infrastructure, bioinformatic expertise, and significant cost [15,41,42]. Our study highlights that phenotypic profiles, particularly resistance to macrolides like erythromycin and azithromycin, can predict MDR status in *Campylobacter* isolates. Since macrolides are considered the first-line antimicrobial class for the treatment of human *Campylobacter*iosis [43], early screening for macrolide resistance not only plays an essential role in guiding antimicrobial therapy but can also serve as an important indicator for MDR prediction in *Campylobacter* spp. isolates, at least those of swine origin.

Resistance to tetracycline and fluoroquinolone is commonly observed in *Campylobacter* isolates [12]; however, based on our findings, these resistances do not always appear to be associated with MDR phenotype, particularly when compared to macrolide resistance. Our results showed that, at least in US swine *Campylobacter* isolates, macrolide resistance may be more consistently associated with broader resistance patterns and may play a more significant role in MDR phenotype. It seems that there is a possibility that some *Campylobacter* isolates exhibit resistance to only tetracycline or fluoroquinolone without concurrent resistance to other antimicrobial classes, and therefore do not meet the criteria for MDR. Further studies are required to elucidate the underlying reasons for this phenomenon.

Compared to our findings, a recent machine learning study on *Campylobacter* spp. reported that ciprofloxacin and tetracycline were among the highest performing classifiers in both *C. coli* and *C. jejuni* isolates [26], which underscores the potential role of the pathogen source and geographical region in determining influential resistance predictors among *Campylobacter* isolates. It should be noted that comparing our findings with similar previous investigations should be treated with caution due to the differences in study design, pathogen’s host, and geographical region. Our findings may also complement genomic research and agree with previous genomic and phenotypic concordance studies [44,45].

Our results also showed that clindamycin resistance (a lincosamide) was among the most important features in predicting MDR status in both training and external validation phases. This shows the importance of considering lincosamide resistance in addition to macrolide resistance in livestock production, as both antimicrobial classes target the bacterial ribosome, particularly the 50S subunit, to inhibit protein synthesis [46].

Compared to the previous similar studies, the power of our study is its two-phase temporal validation design, which addresses a common limitation in machine learning predictive AMR models. By training the model on data from 2013 to 2019 and validating on 2020–2023 (excluding 2022) data, we tried to evaluate real-world generalizability, and we showed our model’s ability to maintain high predictive accuracy in different periods of time. Despite possible shifts in antimicrobial use, biosecurity practices at swine farms, and the emergence of new resistance mechanisms over time, our trained model correctly predicted MDR status in the majority of *Campylobacter* spp. isolates. Of 603 *Campylobacter* spp. isolates in the external validation dataset, only nine MDR isolates were misclassified as non-MDR (93.43% sensitivity), resulting in a few false negatives.

Our model might have potential practical applications, beyond its performance metrics. In resource-limited settings, the model may detect potential MDR isolates based on partial antimicrobial susceptibility results, particularly when macrolide resistance is observed. Such early detection could support targeted interventions in mitigating AMR and MDR in livestock production systems.

Added to that, integrating this type of machine learning model into laboratory data systems has the potential to simplify and speed up the detection of MDR isolates. Automated MDR prediction could be a valuable tool for public health authorities, helping them to detect emerging MDR clusters more quickly and allocate resources more efficiently [47], without the need for panel antimicrobial susceptibility testing or genomic sequencing. This approach can save time, money, and effort. Further machine learning research on AMR surveillance data over time is required to reinforce our findings and shed more light on the broader applicability and potential limitations of phenotypic-based machine learning MDR prediction.

In the present study, we did not conduct species-level modeling (*C. jejuni* vs. *C. coli*) because the dataset was largely composed of *C. coli*, which is the main *Campylobacter* species associated with swine. This imbalance made it difficult to perform meaningful comparisons, so our predictions mainly reflect resistance patterns in *C. coli*. Further studies are required to investigate species-level modeling, where both *C. jejuni* and *C. coli* are more balanced representations, such as *Campylobacter* species isolated from poultry. This may provide more reasonable comparative insights.

This study has several limitations. Our model was trained and validated only on *Campylobacter* spp. isolates from swine in the United States, which may limit its generalizability for predicting MDR in *Campylobacter* spp. from other hosts or geographical regions. Moreover, our model was trained solely on phenotypic resistance data and did not include genomic information, which could influence predictive accuracy and mechanistic interpretability.

## 5. Conclusions

In conclusion, our study presented a robust, interpretable, and temporally validated supervised machine learning model (Random Forest) that indicated high accuracy in predicting MDR status in *Campylobacter* isolates from US swine by using predictors of phenotypic resistance profiles of seven antimicrobials. This model may offer a scalable, cost-effective, and practical complement to traditional phenotypic testing, facilitating the rapid detection and monitoring of MDR in *Campylobacter* spp., particularly through key resistance predictors, like macrolides. Such models or similar approaches may also have potential for real-time implementation in AMR surveillance efforts and AMR diagnostic laboratories. Further validation on different host species and geographic regions, as well as integration of genomic data, will be essential to enhance the model’s generalizability and predictive accuracy in the global battle against antimicrobial resistance in major foodborne pathogens.

## Figures and Tables

**Table 1:** Performance statistics of the trained Random Forest model for predicting MDR in *Campylobacter* isolates from swine.

Metric	Value
Accuracy	99.73%
95% Confidence Interval	99.42–99.90%
No Information Rate (NIR)	76.54%
*p*-Value [Accuracy > NIR]	*p* < 2 × 10^−16^
Kappa	0.9925
McNemar’s Test *p*-Value	0.0412
Sensitivity	98.86%
Specificity	100.00%
Positive Predictive Value	100.00%
Negative Predictive Value	99.65%
Prevalence	23.46%
Detection Rate	23.19%
Detection Prevalence	23.19%
Balanced Accuracy	99.43%

**Table 2:** Performance statistics of the external validation of the Random Forest model for predicting MDR in *Campylobacter* isolates from swine.

Metric	Value
Accuracy	98.51%
95% Confidence Interval	97.19–99.32%
No Information Rate (NIR)	77.28%
*p*-Value [Acc > NIR]	*p* < 2.2 × 10^−16^
Kappa	0.9565
McNemar’s Test *p*-Value	0.007661
Sensitivity	93.43%
Specificity	100%
Positive Predictive Value	100%
Negative Predictive Value	98.11
Prevalence	22.72%
Detection Rate	21.23%
Detection Prevalence	21.%23
Balanced Accuracy	96.72%

## Data Availability

The data is publicly available, the original contributions presented in this study are included in the article/Supplementary Material.

## Supplementary Materials

The following supporting information can be downloaded at: https://www.mdpi.com/article/10.3390/vetsci12100937/s1, Table S1. Performance comparison of five machine learning algorithms for predicting multidrug resistance in swine-derived *Campylobacter* spp. (Accuracy and Kappa); Table S2. Prediction results of the Random Forest model for multidrug resistance in *Campylobacter* isolates from swine (Actual vs. Predicted); Table S3. Prediction results of the external validation phase (Phase 2) of the Random Forest model for multidrug resistance in *Campylobacter* isolates from swine (Actual vs. Predicted).
