# Supplementary material for: Machine Learning Prediction of Multidrug Resistance in Swine-Derived Campylobacter spp. Using United States Antimicrobial Resistance Surveillance Data (2013–2023)
Source: Vet Sci. 2025 Sep 26;12(10):937. doi: 10.3390/vetsci12100937 (PMC12567682; doi:10.3390/vetsci12100937)
Supplement: Supplementary file 1 [file vetsci-12-00937-s001.zip › vetsci-3876008-supplementary.pdf]

Supplementary Table S1. Performance comparison of five machine learning algorithms for predicting multidrug resistance in swine-derived *Campylobacter* spp. (Accuracy and Kappa).

Algorithm	Accuracy.Min.	Accuracy.1st.Qu.	Accuracy.Median	Accuracy.Mean	Accuracy.3rd.Qu.	Accuracy.Max.	Accuracy.NA.s
SVM	0.9933	0.9978	1.0000	0.9982	1.0000	1.0000	0.0000
Decision Tree	0.9711	0.9756	0.9844	0.9831	0.9911	0.9933	0.0000
Random Forest	0.9955	0.9978	1.0000	0.9987	1.0000	1.0000	0.0000
Naive Bayes	0.9579	0.9756	0.9800	0.9796	0.9844	1.0000	0.0000
KNN	0.9933	0.9933	0.9956	0.9956	0.9956	1.0000	0.0000

Algorithm	Kappa.Min.	Kappa.1st.Qu.	Kappa.Median	Kappa.Mean	Kappa.3rd.Qu.	Kappa.Max.	Kappa.NA.s
SVM	0.9814	0.9938	1.0000	0.9950	1.0000	1.0000	0.0000
Decision Tree	0.9162	0.9301	0.9569	0.9520	0.9753	0.9814	0.0000
Random Forest	0.9875	0.9938	1.0000	0.9963	1.0000	1.0000	0.0000
Naive Bayes	0.8840	0.9296	0.9424	0.9424	0.9558	1.0000	0.0000
KNN	0.9814	0.9814	0.9877	0.9877	0.9877	1.0000	0.0000

Supplementary Table S2. Prediction results of the Random Forest model for multidrug resistance in *Campylobacter* isolates from swine (Actual vs. Predicted)

No.	Predicted	Actual	Prediction result	No.	Predicted	Actual	Prediction result
1	N	N	Correct	1127	N	N	Correct
2	N	N	Correct	1128	N	N	Correct
3	N	N	Correct	1129	N	N	Correct
4	N	N	Correct	1130	N	N	Correct
5	N	N	Correct	1131	N	N	Correct
6	N	N	Correct	1132	N	N	Correct
7	N	N	Correct	1133	N	N	Correct
8	N	N	Correct	1134	N	N	Correct
9	N	N	Correct	1135	N	N	Correct
10	N	N	Correct	1136	N	N	Correct
11	N	N	Correct	1137	N	N	Correct
12	N	N	Correct	1138	N	N	Correct
13	N	N	Correct	1139	N	N	Correct
14	N	N	Correct	1140	N	N	Correct
15	N	N	Correct	1141	N	N	Correct
16	N	N	Correct	1142	N	N	Correct
17	N	N	Correct	1143	N	N	Correct
18	N	N	Correct	1144	N	N	Correct
19	N	N	Correct	1145	Y	Y	Correct
20	N	N	Correct	1146	Y	Y	Correct
21	N	N	Correct	1147	Y	Y	Correct
22	N	N	Correct	1148	Y	Y	Correct
23	N	N	Correct	1149	Y	Y	Correct
24	N	N	Correct	1150	Y	Y	Correct
25	N	N	Correct	1151	Y	Y	Correct
26	N	N	Correct	1152	Y	Y	Correct
27	N	N	Correct	1153	Y	Y	Correct
28	N	N	Correct	1154	Y	Y	Correct
29	N	N	Correct	1155	Y	Y	Correct
30	N	N	Correct	1156	Y	Y	Correct
31	N	N	Correct	1157	N	N	Correct
32	N	N	Correct	1158	Y	Y	Correct
33	N	N	Correct	1159	Y	Y	Correct
34	N	N	Correct	1160	Y	Y	Correct
35	N	N	Correct	1161	N	N	Correct
36	N	N	Correct	1162	Y	Y	Correct

37	N	N	Correct	1163	N	N	Correct
38	N	N	Correct	1164	N	N	Correct
39	N	N	Correct	1165	N	N	Correct
40	N	N	Correct	1166	N	N	Correct
41	N	N	Correct	1167	N	N	Correct
42	N	N	Correct	1168	N	N	Correct
43	Y	Y	Correct	1169	N	N	Correct
44	Y	Y	Correct	1170	N	N	Correct
45	Y	Y	Correct	1171	N	N	Correct
46	Y	Y	Correct	1172	N	N	Correct
47	N	N	Correct	1173	N	N	Correct
48	Y	Y	Correct	1174	N	N	Correct
49	Y	Y	Correct	1175	N	N	Correct
50	Y	Y	Correct	1176	N	N	Correct
51	N	N	Correct	1177	N	N	Correct
52	Y	Y	Correct	1178	N	N	Correct
53	Y	Y	Correct	1179	N	N	Correct
54	Y	Y	Correct	1180	N	N	Correct
55	Y	Y	Correct	1181	N	N	Correct
56	N	N	Correct	1182	N	N	Correct
57	Y	Y	Correct	1183	N	N	Correct
58	N	N	Correct	1184	N	N	Correct
59	N	N	Correct	1185	N	N	Correct
60	Y	Y	Correct	1186	N	N	Correct
61	N	N	Correct	1187	N	N	Correct
62	N	N	Correct	1188	N	N	Correct
63	N	N	Correct	1189	N	N	Correct
64	N	N	Correct	1190	N	N	Correct
65	N	N	Correct	1191	N	N	Correct
66	N	N	Correct	1192	N	N	Correct
67	N	N	Correct	1193	N	N	Correct
68	N	N	Correct	1194	N	N	Correct
69	N	N	Correct	1195	N	N	Correct
70	N	N	Correct	1196	N	N	Correct
71	N	N	Correct	1197	N	N	Correct
72	N	N	Correct	1198	N	N	Correct
73	N	N	Correct	1199	N	N	Correct
74	N	N	Correct	1200	N	N	Correct
75	N	N	Correct	1201	N	N	Correct
76	N	N	Correct	1202	N	N	Correct
77	N	N	Correct	1203	N	N	Correct

78	N	N	Correct	1204	N	N	Correct
79	N	N	Correct	1205	N	N	Correct
80	N	N	Correct	1206	N	N	Correct
81	N	N	Correct	1207	N	N	Correct
82	N	N	Correct	1208	N	N	Correct
83	N	N	Correct	1209	N	N	Correct
84	N	N	Correct	1210	N	N	Correct
85	N	N	Correct	1211	N	N	Correct
86	N	N	Correct	1212	Y	Y	Correct
87	N	N	Correct	1213	Y	Y	Correct
88	N	N	Correct	1214	Y	Y	Correct
89	N	N	Correct	1215	Y	Y	Correct
90	N	N	Correct	1216	Y	Y	Correct
91	N	N	Correct	1217	Y	Y	Correct
92	N	N	Correct	1218	Y	Y	Correct
93	N	N	Correct	1219	N	N	Correct
94	N	N	Correct	1220	Y	Y	Correct
95	N	N	Correct	1221	Y	Y	Correct
96	N	N	Correct	1222	N	N	Correct
97	N	N	Correct	1223	N	N	Correct
98	Y	Y	Correct	1224	N	N	Correct
99	Y	Y	Correct	1225	N	N	Correct
100	Y	Y	Correct	1226	N	N	Correct
101	Y	Y	Correct	1227	N	N	Correct
102	Y	Y	Correct	1228	N	N	Correct
103	Y	Y	Correct	1229	Y	Y	Correct
104	Y	Y	Correct	1230	Y	Y	Correct
105	Y	Y	Correct	1231	Y	Y	Correct
106	Y	Y	Correct	1232	Y	Y	Correct
107	N	N	Correct	1233	Y	Y	Correct
108	N	N	Correct	1234	Y	Y	Correct
109	N	N	Correct	1235	N	N	Correct
110	N	N	Correct	1236	N	N	Correct
111	Y	Y	Correct	1237	N	N	Correct
112	Y	Y	Correct	1238	N	N	Correct
113	Y	Y	Correct	1239	Y	Y	Correct
114	Y	Y	Correct	1240	Y	Y	Correct
115	Y	Y	Correct	1241	Y	Y	Correct
116	Y	Y	Correct	1242	Y	Y	Correct
117	Y	Y	Correct	1243	N	N	Correct
118	Y	Y	Correct	1244	N	N	Correct

119	Y	Y	Correct	1245	N	N	Correct
120	Y	Y	Correct	1246	N	N	Correct
121	Y	Y	Correct	1247	N	N	Correct
122	N	N	Correct	1248	N	N	Correct
123	N	N	Correct	1249	N	N	Correct
124	N	N	Correct	1250	N	N	Correct
125	N	N	Correct	1251	N	N	Correct
126	N	N	Correct	1252	N	N	Correct
127	N	N	Correct	1253	N	N	Correct
128	N	N	Correct	1254	N	N	Correct
129	N	N	Correct	1255	N	N	Correct
130	N	N	Correct	1256	N	N	Correct
131	N	N	Correct	1257	N	N	Correct
132	N	N	Correct	1258	N	N	Correct
133	N	N	Correct	1259	N	N	Correct
134	N	N	Correct	1260	N	N	Correct
135	N	N	Correct	1261	N	N	Correct
136	N	N	Correct	1262	N	N	Correct
137	N	N	Correct	1263	N	N	Correct
138	N	N	Correct	1264	N	N	Correct
139	N	N	Correct	1265	N	N	Correct
140	N	N	Correct	1266	N	N	Correct
141	N	N	Correct	1267	N	N	Correct
142	N	N	Correct	1268	N	N	Correct
143	N	N	Correct	1269	N	N	Correct
144	N	N	Correct	1270	N	N	Correct
145	N	N	Correct	1271	N	N	Correct
146	N	N	Correct	1272	N	N	Correct
147	N	N	Correct	1273	N	N	Correct
148	N	N	Correct	1274	N	N	Correct
149	N	N	Correct	1275	N	N	Correct
150	N	N	Correct	1276	N	N	Correct
151	N	N	Correct	1277	N	N	Correct
152	N	N	Correct	1278	N	N	Correct
153	N	N	Correct	1279	N	N	Correct
154	N	N	Correct	1280	N	N	Correct
155	N	N	Correct	1281	N	N	Correct
156	N	N	Correct	1282	N	N	Correct
157	N	N	Correct	1283	Y	Y	Correct
158	N	N	Correct	1284	Y	Y	Correct
159	N	N	Correct	1285	Y	Y	Correct

160	N	N	Correct	1286	Y	Y	Correct
161	N	N	Correct	1287	Y	Y	Correct
162	N	N	Correct	1288	Y	Y	Correct
163	N	N	Correct	1289	Y	Y	Correct
164	N	N	Correct	1290	Y	Y	Correct
165	N	N	Correct	1291	Y	Y	Correct
166	N	N	Correct	1292	Y	Y	Correct
167	N	N	Correct	1293	Y	Y	Correct
168	N	N	Correct	1294	Y	Y	Correct
169	N	N	Correct	1295	Y	Y	Correct
170	N	N	Correct	1296	Y	Y	Correct
171	N	N	Correct	1297	Y	Y	Correct
172	N	N	Correct	1298	Y	Y	Correct
173	N	N	Correct	1299	Y	Y	Correct
174	Y	Y	Correct	1300	N	N	Correct
175	Y	Y	Correct	1301	N	N	Correct
176	Y	Y	Correct	1302	N	N	Correct
177	Y	Y	Correct	1303	N	N	Correct
178	N	N	Correct	1304	N	N	Correct
179	N	N	Correct	1305	N	N	Correct
180	N	N	Correct	1306	N	N	Correct
181	N	N	Correct	1307	N	N	Correct
182	N	N	Correct	1308	N	N	Correct
183	N	N	Correct	1309	N	N	Correct
184	N	N	Correct	1310	N	N	Correct
185	N	N	Correct	1311	N	N	Correct
186	Y	Y	Correct	1312	N	N	Correct
187	N	N	Correct	1313	N	N	Correct
188	Y	Y	Correct	1314	N	N	Correct
189	N	N	Correct	1315	N	N	Correct
190	N	N	Correct	1316	N	N	Correct
191	N	N	Correct	1317	N	N	Correct
192	N	N	Correct	1318	N	N	Correct
193	N	N	Correct	1319	N	N	Correct
194	N	N	Correct	1320	N	N	Correct
195	Y	Y	Correct	1321	N	N	Correct
196	N	N	Correct	1322	N	N	Correct
197	Y	Y	Correct	1323	N	N	Correct
198	Y	Y	Correct	1324	N	N	Correct
199	Y	Y	Correct	1325	N	N	Correct
200	Y	Y	Correct	1326	N	N	Correct

201	Y	Y	Correct	1327	N	N	Correct
202	Y	Y	Correct	1328	N	N	Correct
203	Y	Y	Correct	1329	N	N	Correct
204	Y	Y	Correct	1330	N	N	Correct
205	Y	Y	Correct	1331	N	N	Correct
206	Y	Y	Correct	1332	N	N	Correct
207	Y	Y	Correct	1333	N	N	Correct
208	Y	Y	Correct	1334	N	N	Correct
209	Y	Y	Correct	1335	N	N	Correct
210	Y	Y	Correct	1336	N	N	Correct
211	Y	Y	Correct	1337	N	N	Correct
212	Y	Y	Correct	1338	N	N	Correct
213	N	N	Correct	1339	N	N	Correct
214	N	N	Correct	1340	N	N	Correct
215	N	N	Correct	1341	N	N	Correct
216	N	N	Correct	1342	N	N	Correct
217	N	N	Correct	1343	N	N	Correct
218	N	N	Correct	1344	N	N	Correct
219	N	N	Correct	1345	N	N	Correct
220	N	N	Correct	1346	N	N	Correct
221	N	N	Correct	1347	N	N	Correct
222	N	N	Correct	1348	N	N	Correct
223	N	N	Correct	1349	N	N	Correct
224	N	N	Correct	1350	N	N	Correct
225	N	N	Correct	1351	N	N	Correct
226	N	N	Correct	1352	N	N	Correct
227	N	N	Correct	1353	N	N	Correct
228	N	N	Correct	1354	N	N	Correct
229	N	N	Correct	1355	N	N	Correct
230	N	N	Correct	1356	N	N	Correct
231	N	N	Correct	1357	N	N	Correct
232	N	N	Correct	1358	N	N	Correct
233	N	N	Correct	1359	N	N	Correct
234	N	N	Correct	1360	N	N	Correct
235	N	N	Correct	1361	N	N	Correct
236	N	N	Correct	1362	N	N	Correct
237	N	N	Correct	1363	N	N	Correct
238	N	N	Correct	1364	N	N	Correct
239	N	N	Correct	1365	N	N	Correct
240	N	N	Correct	1366	N	N	Correct
241	N	N	Correct	1367	N	N	Correct

242	N	N	Correct	1368	N	N	Correct
243	N	N	Correct	1369	N	N	Correct
244	N	N	Correct	1370	N	N	Correct
245	N	Y	Mismatch	1371	N	N	Correct
246	N	N	Correct	1372	N	N	Correct
247	N	N	Correct	1373	N	N	Correct
248	N	N	Correct	1374	N	N	Correct
249	N	N	Correct	1375	N	N	Correct
250	N	N	Correct	1376	N	N	Correct
251	N	N	Correct	1377	N	N	Correct
252	N	N	Correct	1378	N	N	Correct
253	N	N	Correct	1379	N	N	Correct
254	N	N	Correct	1380	N	N	Correct
255	N	N	Correct	1381	N	N	Correct
256	N	N	Correct	1382	N	N	Correct
257	N	N	Correct	1383	N	N	Correct
258	N	N	Correct	1384	N	N	Correct
259	N	N	Correct	1385	N	N	Correct
260	N	N	Correct	1386	N	N	Correct
261	N	N	Correct	1387	N	N	Correct
262	N	N	Correct	1388	N	N	Correct
263	N	N	Correct	1389	N	N	Correct
264	N	N	Correct	1390	Y	Y	Correct
265	N	N	Correct	1391	Y	Y	Correct
266	N	N	Correct	1392	Y	Y	Correct
267	N	N	Correct	1393	N	N	Correct
268	N	N	Correct	1394	Y	Y	Correct
269	N	N	Correct	1395	Y	Y	Correct
270	N	N	Correct	1396	Y	Y	Correct
271	Y	Y	Correct	1397	N	N	Correct
272	N	N	Correct	1398	N	N	Correct
273	Y	Y	Correct	1399	Y	Y	Correct
274	Y	Y	Correct	1400	Y	Y	Correct
275	N	N	Correct	1401	Y	Y	Correct
276	Y	Y	Correct	1402	N	N	Correct
277	N	N	Correct	1403	Y	Y	Correct
278	Y	Y	Correct	1404	Y	Y	Correct
279	Y	Y	Correct	1405	Y	Y	Correct
280	Y	Y	Correct	1406	Y	Y	Correct
281	Y	Y	Correct	1407	Y	Y	Correct
282	Y	Y	Correct	1408	N	N	Correct

283	Y	Y	Correct	1409	N	N	Correct
284	Y	Y	Correct	1410	N	N	Correct
285	Y	Y	Correct	1411	N	N	Correct
286	Y	Y	Correct	1412	N	N	Correct
287	Y	Y	Correct	1413	N	N	Correct
288	Y	Y	Correct	1414	N	N	Correct
289	Y	Y	Correct	1415	N	N	Correct
290	N	N	Correct	1416	N	N	Correct
291	N	N	Correct	1417	N	N	Correct
292	N	N	Correct	1418	N	N	Correct
293	N	N	Correct	1419	N	N	Correct
294	N	N	Correct	1420	N	N	Correct
295	N	N	Correct	1421	N	N	Correct
296	N	N	Correct	1422	N	N	Correct
297	N	N	Correct	1423	N	N	Correct
298	N	N	Correct	1424	N	N	Correct
299	N	N	Correct	1425	N	N	Correct
300	N	N	Correct	1426	N	N	Correct
301	N	N	Correct	1427	N	N	Correct
302	N	N	Correct	1428	N	N	Correct
303	N	N	Correct	1429	N	N	Correct
304	N	N	Correct	1430	N	N	Correct
305	N	N	Correct	1431	N	N	Correct
306	N	N	Correct	1432	N	N	Correct
307	N	N	Correct	1433	N	N	Correct
308	N	N	Correct	1434	N	N	Correct
309	N	N	Correct	1435	N	N	Correct
310	N	N	Correct	1436	N	N	Correct
311	N	N	Correct	1437	N	N	Correct
312	N	N	Correct	1438	N	N	Correct
313	N	N	Correct	1439	N	N	Correct
314	N	N	Correct	1440	N	N	Correct
315	N	N	Correct	1441	N	N	Correct
316	N	N	Correct	1442	N	N	Correct
317	N	N	Correct	1443	N	N	Correct
318	Y	Y	Correct	1444	N	N	Correct
319	N	N	Correct	1445	N	N	Correct
320	N	N	Correct	1446	N	N	Correct
321	N	N	Correct	1447	N	N	Correct
322	N	N	Correct	1448	N	N	Correct
323	Y	Y	Correct	1449	Y	Y	Correct

324	Y	Y	Correct	1450	Y	Y	Correct
325	N	N	Correct	1451	Y	Y	Correct
326	Y	Y	Correct	1452	Y	Y	Correct
327	Y	Y	Correct	1453	Y	Y	Correct
328	Y	Y	Correct	1454	Y	Y	Correct
329	N	N	Correct	1455	Y	Y	Correct
330	N	N	Correct	1456	Y	Y	Correct
331	N	N	Correct	1457	Y	Y	Correct
332	N	N	Correct	1458	Y	Y	Correct
333	N	N	Correct	1459	Y	Y	Correct
334	N	N	Correct	1460	N	N	Correct
335	N	N	Correct	1461	Y	Y	Correct
336	Y	Y	Correct	1462	Y	Y	Correct
337	Y	Y	Correct	1463	Y	Y	Correct
338	Y	Y	Correct	1464	Y	Y	Correct
339	Y	Y	Correct	1465	Y	Y	Correct
340	Y	Y	Correct	1466	N	N	Correct
341	Y	Y	Correct	1467	N	N	Correct
342	Y	Y	Correct	1468	N	N	Correct
343	N	N	Correct	1469	N	N	Correct
344	Y	Y	Correct	1470	N	N	Correct
345	N	N	Correct	1471	N	N	Correct
346	N	N	Correct	1472	Y	Y	Correct
347	N	N	Correct	1473	Y	Y	Correct
348	N	N	Correct	1474	Y	Y	Correct
349	N	N	Correct	1475	Y	Y	Correct
350	N	N	Correct	1476	Y	Y	Correct
351	N	N	Correct	1477	Y	Y	Correct
352	N	N	Correct	1478	Y	Y	Correct
353	Y	Y	Correct	1479	N	N	Correct
354	Y	Y	Correct	1480	N	N	Correct
355	N	N	Correct	1481	N	N	Correct
356	N	N	Correct	1482	N	N	Correct
357	N	N	Correct	1483	N	N	Correct
358	N	N	Correct	1484	N	N	Correct
359	N	N	Correct	1485	N	N	Correct
360	N	N	Correct	1486	N	N	Correct
361	N	N	Correct	1487	N	N	Correct
362	N	N	Correct	1488	N	N	Correct
363	N	N	Correct	1489	N	N	Correct
364	N	N	Correct	1490	N	N	Correct

365	N	N	Correct	1491	N	N	Correct
366	N	N	Correct	1492	N	N	Correct
367	N	N	Correct	1493	N	N	Correct
368	N	N	Correct	1494	N	N	Correct
369	N	N	Correct	1495	N	N	Correct
370	N	N	Correct	1496	N	N	Correct
371	N	N	Correct	1497	N	N	Correct
372	N	N	Correct	1498	N	N	Correct
373	N	N	Correct	1499	N	N	Correct
374	N	N	Correct	1500	N	N	Correct
375	N	N	Correct	1501	N	N	Correct
376	N	N	Correct	1502	N	N	Correct
377	N	N	Correct	1503	N	N	Correct
378	N	N	Correct	1504	N	N	Correct
379	N	N	Correct	1505	N	N	Correct
380	N	N	Correct	1506	N	N	Correct
381	N	N	Correct	1507	N	N	Correct
382	N	N	Correct	1508	N	N	Correct
383	N	N	Correct	1509	N	N	Correct
384	N	N	Correct	1510	N	N	Correct
385	Y	Y	Correct	1511	N	N	Correct
386	Y	Y	Correct	1512	N	N	Correct
387	Y	Y	Correct	1513	N	N	Correct
388	Y	Y	Correct	1514	N	N	Correct
389	Y	Y	Correct	1515	N	N	Correct
390	Y	Y	Correct	1516	N	N	Correct
391	Y	Y	Correct	1517	N	N	Correct
392	Y	Y	Correct	1518	N	N	Correct
393	Y	Y	Correct	1519	N	N	Correct
394	Y	Y	Correct	1520	N	N	Correct
395	Y	Y	Correct	1521	N	N	Correct
396	N	N	Correct	1522	N	N	Correct
397	Y	Y	Correct	1523	N	N	Correct
398	Y	Y	Correct	1524	N	N	Correct
399	Y	Y	Correct	1525	N	N	Correct
400	Y	Y	Correct	1526	N	N	Correct
401	Y	Y	Correct	1527	N	N	Correct
402	Y	Y	Correct	1528	N	N	Correct
403	N	N	Correct	1529	N	N	Correct
404	N	N	Correct	1530	N	N	Correct
405	N	N	Correct	1531	Y	Y	Correct

406	N	N	Correct	1532	Y	Y	Correct
407	N	N	Correct	1533	Y	Y	Correct
408	N	N	Correct	1534	Y	Y	Correct
409	N	N	Correct	1535	Y	Y	Correct
410	N	N	Correct	1536	Y	Y	Correct
411	N	N	Correct	1537	N	N	Correct
412	N	N	Correct	1538	Y	Y	Correct
413	N	N	Correct	1539	N	N	Correct
414	N	N	Correct	1540	N	N	Correct
415	N	N	Correct	1541	N	N	Correct
416	N	N	Correct	1542	N	N	Correct
417	N	N	Correct	1543	N	N	Correct
418	N	N	Correct	1544	N	N	Correct
419	N	N	Correct	1545	N	N	Correct
420	N	N	Correct	1546	N	N	Correct
421	N	N	Correct	1547	N	N	Correct
422	N	N	Correct	1548	Y	Y	Correct
423	N	N	Correct	1549	Y	Y	Correct
424	N	N	Correct	1550	Y	Y	Correct
425	N	N	Correct	1551	N	N	Correct
426	N	N	Correct	1552	Y	Y	Correct
427	N	N	Correct	1553	Y	Y	Correct
428	N	N	Correct	1554	N	N	Correct
429	N	N	Correct	1555	N	N	Correct
430	N	N	Correct	1556	N	N	Correct
431	N	N	Correct	1557	N	N	Correct
432	N	N	Correct	1558	N	N	Correct
433	N	N	Correct	1559	N	N	Correct
434	N	N	Correct	1560	N	N	Correct
435	N	N	Correct	1561	N	N	Correct
436	N	N	Correct	1562	N	N	Correct
437	N	N	Correct	1563	N	N	Correct
438	N	N	Correct	1564	N	N	Correct
439	N	N	Correct	1565	N	N	Correct
440	N	N	Correct	1566	Y	Y	Correct
441	N	N	Correct	1567	Y	Y	Correct
442	N	N	Correct	1568	Y	Y	Correct
443	N	N	Correct	1569	Y	Y	Correct
444	N	N	Correct	1570	Y	Y	Correct
445	N	N	Correct	1571	Y	Y	Correct
446	N	N	Correct	1572	Y	Y	Correct

447	N	N	Correct	1573	Y	Y	Correct
448	N	N	Correct	1574	Y	Y	Correct
449	N	N	Correct	1575	Y	Y	Correct
450	N	N	Correct	1576	Y	Y	Correct
451	N	N	Correct	1577	Y	Y	Correct
452	N	N	Correct	1578	Y	Y	Correct
453	N	N	Correct	1579	N	N	Correct
454	N	N	Correct	1580	N	N	Correct
455	N	N	Correct	1581	N	N	Correct
456	N	N	Correct	1582	N	N	Correct
457	N	N	Correct	1583	N	N	Correct
458	N	N	Correct	1584	N	N	Correct
459	N	N	Correct	1585	N	N	Correct
460	N	N	Correct	1586	N	N	Correct
461	N	N	Correct	1587	N	N	Correct
462	N	N	Correct	1588	N	N	Correct
463	N	N	Correct	1589	N	N	Correct
464	N	N	Correct	1590	N	N	Correct
465	N	N	Correct	1591	N	N	Correct
466	N	N	Correct	1592	N	N	Correct
467	N	N	Correct	1593	N	N	Correct
468	N	N	Correct	1594	N	N	Correct
469	N	N	Correct	1595	N	N	Correct
470	N	N	Correct	1596	N	N	Correct
471	N	N	Correct	1597	N	N	Correct
472	N	N	Correct	1598	N	N	Correct
473	N	N	Correct	1599	N	N	Correct
474	N	N	Correct	1600	N	N	Correct
475	N	N	Correct	1601	N	N	Correct
476	N	N	Correct	1602	N	N	Correct
477	N	N	Correct	1603	N	N	Correct
478	N	N	Correct	1604	N	N	Correct
479	N	N	Correct	1605	N	N	Correct
480	N	N	Correct	1606	N	N	Correct
481	N	N	Correct	1607	N	N	Correct
482	N	N	Correct	1608	N	N	Correct
483	N	N	Correct	1609	N	N	Correct
484	N	N	Correct	1610	N	N	Correct
485	N	N	Correct	1611	N	N	Correct
486	N	N	Correct	1612	N	N	Correct
487	N	N	Correct	1613	N	N	Correct

488	N	N	Correct	1614	N	N	Correct
489	N	N	Correct	1615	N	N	Correct
490	N	N	Correct	1616	N	N	Correct
491	N	N	Correct	1617	N	N	Correct
492	N	N	Correct	1618	N	N	Correct
493	N	N	Correct	1619	N	N	Correct
494	N	N	Correct	1620	Y	Y	Correct
495	N	N	Correct	1621	N	N	Correct
496	N	N	Correct	1622	N	N	Correct
497	N	N	Correct	1623	N	N	Correct
498	N	N	Correct	1624	N	N	Correct
499	Y	Y	Correct	1625	Y	Y	Correct
500	N	N	Correct	1626	Y	Y	Correct
501	Y	Y	Correct	1627	N	N	Correct
502	Y	Y	Correct	1628	Y	Y	Correct
503	Y	Y	Correct	1629	Y	Y	Correct
504	Y	Y	Correct	1630	Y	Y	Correct
505	Y	Y	Correct	1631	Y	Y	Correct
506	Y	Y	Correct	1632	Y	Y	Correct
507	Y	Y	Correct	1633	Y	Y	Correct
508	Y	Y	Correct	1634	Y	Y	Correct
509	Y	Y	Correct	1635	Y	Y	Correct
510	Y	Y	Correct	1636	Y	Y	Correct
511	Y	Y	Correct	1637	N	N	Correct
512	Y	Y	Correct	1638	Y	Y	Correct
513	Y	Y	Correct	1639	Y	Y	Correct
514	Y	Y	Correct	1640	Y	Y	Correct
515	N	N	Correct	1641	Y	Y	Correct
516	Y	Y	Correct	1642	N	N	Correct
517	Y	Y	Correct	1643	N	N	Correct
518	N	N	Correct	1644	N	N	Correct
519	N	N	Correct	1645	N	N	Correct
520	N	N	Correct	1646	N	N	Correct
521	N	N	Correct	1647	N	N	Correct
522	N	N	Correct	1648	N	N	Correct
523	N	N	Correct	1649	N	N	Correct
524	N	N	Correct	1650	N	N	Correct
525	N	N	Correct	1651	N	N	Correct
526	N	N	Correct	1652	N	N	Correct
527	N	N	Correct	1653	N	N	Correct
528	N	N	Correct	1654	N	N	Correct

529	N	N	Correct	1655	N	N	Correct
530	N	N	Correct	1656	N	N	Correct
531	N	N	Correct	1657	N	N	Correct
532	N	N	Correct	1658	N	N	Correct
533	N	N	Correct	1659	N	N	Correct
534	N	N	Correct	1660	N	N	Correct
535	N	N	Correct	1661	N	N	Correct
536	N	N	Correct	1662	N	N	Correct
537	N	N	Correct	1663	N	N	Correct
538	N	N	Correct	1664	N	N	Correct
539	N	N	Correct	1665	N	N	Correct
540	N	N	Correct	1666	N	N	Correct
541	N	N	Correct	1667	N	N	Correct
542	N	N	Correct	1668	N	N	Correct
543	N	N	Correct	1669	N	N	Correct
544	N	N	Correct	1670	N	N	Correct
545	N	N	Correct	1671	N	N	Correct
546	Y	Y	Correct	1672	N	N	Correct
547	Y	Y	Correct	1673	N	N	Correct
548	Y	Y	Correct	1674	N	N	Correct
549	Y	Y	Correct	1675	N	N	Correct
550	Y	Y	Correct	1676	N	N	Correct
551	Y	Y	Correct	1677	N	N	Correct
552	Y	Y	Correct	1678	N	N	Correct
553	Y	Y	Correct	1679	N	N	Correct
554	N	N	Correct	1680	N	N	Correct
555	Y	Y	Correct	1681	N	N	Correct
556	Y	Y	Correct	1682	N	N	Correct
557	Y	Y	Correct	1683	N	N	Correct
558	Y	Y	Correct	1684	N	N	Correct
559	Y	Y	Correct	1685	Y	Y	Correct
560	Y	Y	Correct	1686	Y	Y	Correct
561	Y	Y	Correct	1687	Y	Y	Correct
562	Y	Y	Correct	1688	Y	Y	Correct
563	Y	Y	Correct	1689	N	N	Correct
564	N	N	Correct	1690	Y	Y	Correct
565	N	N	Correct	1691	Y	Y	Correct
566	N	N	Correct	1692	Y	Y	Correct
567	Y	Y	Correct	1693	N	N	Correct
568	Y	Y	Correct	1694	Y	Y	Correct
569	Y	Y	Correct	1695	N	N	Correct

570	Y	Y	Correct	1696	N	N	Correct
571	N	N	Correct	1697	N	N	Correct
572	N	N	Correct	1698	N	N	Correct
573	N	N	Correct	1699	Y	Y	Correct
574	N	N	Correct	1700	Y	Y	Correct
575	N	N	Correct	1701	Y	Y	Correct
576	N	N	Correct	1702	Y	Y	Correct
577	N	N	Correct	1703	N	N	Correct
578	N	N	Correct	1704	N	N	Correct
579	N	N	Correct	1705	N	N	Correct
580	N	N	Correct	1706	N	N	Correct
581	N	N	Correct	1707	N	N	Correct
582	N	N	Correct	1708	N	N	Correct
583	N	N	Correct	1709	N	N	Correct
584	N	N	Correct	1710	N	N	Correct
585	N	N	Correct	1711	N	N	Correct
586	N	N	Correct	1712	N	N	Correct
587	N	N	Correct	1713	N	N	Correct
588	N	N	Correct	1714	N	N	Correct
589	N	N	Correct	1715	N	N	Correct
590	N	N	Correct	1716	N	N	Correct
591	N	N	Correct	1717	N	N	Correct
592	N	N	Correct	1718	N	N	Correct
593	N	N	Correct	1719	N	N	Correct
594	N	N	Correct	1720	N	N	Correct
595	N	N	Correct	1721	N	N	Correct
596	N	N	Correct	1722	N	N	Correct
597	N	N	Correct	1723	N	N	Correct
598	N	N	Correct	1724	N	N	Correct
599	N	N	Correct	1725	N	N	Correct
600	N	N	Correct	1726	N	N	Correct
601	N	N	Correct	1727	N	N	Correct
602	N	N	Correct	1728	N	N	Correct
603	N	N	Correct	1729	N	N	Correct
604	N	N	Correct	1730	N	N	Correct
605	N	N	Correct	1731	N	N	Correct
606	N	N	Correct	1732	N	N	Correct
607	N	N	Correct	1733	N	N	Correct
608	N	N	Correct	1734	N	N	Correct
609	N	N	Correct	1735	N	N	Correct
610	N	N	Correct	1736	N	N	Correct

611	N	N	Correct	1737	N	N	Correct
612	N	N	Correct	1738	N	N	Correct
613	N	N	Correct	1739	N	N	Correct
614	Y	Y	Correct	1740	N	N	Correct
615	Y	Y	Correct	1741	N	N	Correct
616	Y	Y	Correct	1742	N	N	Correct
617	N	N	Correct	1743	Y	Y	Correct
618	Y	Y	Correct	1744	Y	Y	Correct
619	Y	Y	Correct	1745	Y	Y	Correct
620	Y	Y	Correct	1746	Y	Y	Correct
621	Y	Y	Correct	1747	N	N	Correct
622	Y	Y	Correct	1748	Y	Y	Correct
623	Y	Y	Correct	1749	Y	Y	Correct
624	Y	Y	Correct	1750	Y	Y	Correct
625	N	N	Correct	1751	Y	Y	Correct
626	N	N	Correct	1752	Y	Y	Correct
627	N	N	Correct	1753	Y	Y	Correct
628	N	N	Correct	1754	Y	Y	Correct
629	N	N	Correct	1755	N	N	Correct
630	N	N	Correct	1756	Y	Y	Correct
631	N	N	Correct	1757	Y	Y	Correct
632	N	N	Correct	1758	Y	Y	Correct
633	N	N	Correct	1759	N	N	Correct
634	N	N	Correct	1760	N	N	Correct
635	N	N	Correct	1761	N	N	Correct
636	Y	Y	Correct	1762	N	N	Correct
637	Y	Y	Correct	1763	N	N	Correct
638	N	N	Correct	1764	N	N	Correct
639	Y	Y	Correct	1765	N	N	Correct
640	Y	Y	Correct	1766	N	N	Correct
641	Y	Y	Correct	1767	N	N	Correct
642	N	N	Correct	1768	N	N	Correct
643	N	N	Correct	1769	N	N	Correct
644	N	N	Correct	1770	N	N	Correct
645	N	N	Correct	1771	N	N	Correct
646	N	N	Correct	1772	N	N	Correct
647	N	N	Correct	1773	N	N	Correct
648	N	N	Correct	1774	N	N	Correct
649	N	N	Correct	1775	N	N	Correct
650	N	N	Correct	1776	N	N	Correct
651	Y	Y	Correct	1777	N	N	Correct

652	Y	Y	Correct	1778	N	N	Correct
653	Y	Y	Correct	1779	N	N	Correct
654	Y	Y	Correct	1780	N	N	Correct
655	Y	Y	Correct	1781	N	N	Correct
656	Y	Y	Correct	1782	N	N	Correct
657	Y	Y	Correct	1783	N	N	Correct
658	N	N	Correct	1784	N	Y	Mismatch
659	Y	Y	Correct	1785	N	N	Correct
660	Y	Y	Correct	1786	N	N	Correct
661	Y	Y	Correct	1787	N	N	Correct
662	Y	Y	Correct	1788	N	N	Correct
663	Y	Y	Correct	1789	N	N	Correct
664	N	N	Correct	1790	N	N	Correct
665	Y	Y	Correct	1791	N	N	Correct
666	Y	Y	Correct	1792	N	N	Correct
667	N	N	Correct	1793	N	N	Correct
668	N	N	Correct	1794	N	N	Correct
669	N	N	Correct	1795	N	N	Correct
670	N	N	Correct	1796	N	N	Correct
671	N	N	Correct	1797	N	N	Correct
672	N	N	Correct	1798	N	N	Correct
673	N	N	Correct	1799	N	N	Correct
674	N	N	Correct	1800	N	N	Correct
675	N	N	Correct	1801	N	N	Correct
676	N	N	Correct	1802	N	N	Correct
677	N	N	Correct	1803	N	N	Correct
678	N	N	Correct	1804	N	N	Correct
679	N	N	Correct	1805	N	N	Correct
680	N	N	Correct	1806	N	N	Correct
681	N	N	Correct	1807	N	N	Correct
682	N	N	Correct	1808	N	N	Correct
683	N	N	Correct	1809	N	N	Correct
684	N	N	Correct	1810	N	N	Correct
685	N	N	Correct	1811	N	N	Correct
686	N	N	Correct	1812	N	N	Correct
687	N	N	Correct	1813	N	N	Correct
688	N	N	Correct	1814	N	N	Correct
689	N	N	Correct	1815	N	N	Correct
690	N	N	Correct	1816	N	N	Correct
691	N	N	Correct	1817	N	N	Correct
692	N	N	Correct	1818	N	N	Correct

693	N	N	Correct	1819	N	N	Correct
694	N	N	Correct	1820	N	N	Correct
695	N	N	Correct	1821	N	N	Correct
696	Y	Y	Correct	1822	N	N	Correct
697	N	Y	Mismatch	1823	N	N	Correct
698	N	N	Correct	1824	N	N	Correct
699	Y	Y	Correct	1825	N	N	Correct
700	Y	Y	Correct	1826	N	N	Correct
701	N	N	Correct	1827	N	N	Correct
702	Y	Y	Correct	1828	N	N	Correct
703	Y	Y	Correct	1829	N	N	Correct
704	Y	Y	Correct	1830	N	N	Correct
705	Y	Y	Correct	1831	N	N	Correct
706	Y	Y	Correct	1832	N	N	Correct
707	Y	Y	Correct	1833	N	N	Correct
708	N	N	Correct	1834	N	N	Correct
709	Y	Y	Correct	1835	N	N	Correct
710	N	Y	Mismatch	1836	N	N	Correct
711	N	N	Correct	1837	N	N	Correct
712	N	N	Correct	1838	N	N	Correct
713	N	N	Correct	1839	N	N	Correct
714	N	N	Correct	1840	N	N	Correct
715	N	N	Correct	1841	Y	Y	Correct
716	N	N	Correct	1842	Y	Y	Correct
717	N	N	Correct	1843	Y	Y	Correct
718	N	N	Correct	1844	Y	Y	Correct
719	N	N	Correct	1845	Y	Y	Correct
720	N	N	Correct	1846	Y	Y	Correct
721	N	N	Correct	1847	Y	Y	Correct
722	N	N	Correct	1848	Y	Y	Correct
723	N	N	Correct	1849	Y	Y	Correct
724	N	N	Correct	1850	Y	Y	Correct
725	N	N	Correct	1851	Y	Y	Correct
726	N	N	Correct	1852	Y	Y	Correct
727	N	N	Correct	1853	Y	Y	Correct
728	N	N	Correct	1854	Y	Y	Correct
729	N	N	Correct	1855	Y	Y	Correct
730	N	N	Correct	1856	N	N	Correct
731	N	N	Correct	1857	Y	Y	Correct
732	N	N	Correct	1858	Y	Y	Correct
733	N	N	Correct	1859	Y	Y	Correct

734	N	N	Correct	1860	N	N	Correct
735	N	N	Correct	1861	N	N	Correct
736	N	N	Correct	1862	N	N	Correct
737	N	N	Correct	1863	N	N	Correct
738	N	N	Correct	1864	N	N	Correct
739	N	N	Correct	1865	N	N	Correct
740	N	N	Correct	1866	N	N	Correct
741	N	N	Correct	1867	N	N	Correct
742	N	N	Correct	1868	N	N	Correct
743	N	N	Correct	1869	N	N	Correct
744	N	N	Correct	1870	N	N	Correct
745	N	N	Correct	1871	N	N	Correct
746	N	N	Correct	1872	N	N	Correct
747	N	N	Correct	1873	N	N	Correct
748	N	N	Correct	1874	N	N	Correct
749	N	N	Correct	1875	N	N	Correct
750	N	N	Correct	1876	N	N	Correct
751	N	N	Correct	1877	N	N	Correct
752	N	N	Correct	1878	N	N	Correct
753	N	N	Correct	1879	N	N	Correct
754	N	N	Correct	1880	N	N	Correct
755	N	N	Correct	1881	N	N	Correct
756	Y	Y	Correct	1882	N	N	Correct
757	Y	Y	Correct	1883	N	N	Correct
758	Y	Y	Correct	1884	N	N	Correct
759	Y	Y	Correct	1885	N	N	Correct
760	Y	Y	Correct	1886	N	N	Correct
761	Y	Y	Correct	1887	N	N	Correct
762	N	N	Correct	1888	N	N	Correct
763	N	Y	Mismatch	1889	N	N	Correct
764	N	N	Correct	1890	N	N	Correct
765	N	N	Correct	1891	N	N	Correct
766	N	N	Correct	1892	N	N	Correct
767	N	N	Correct	1893	N	N	Correct
768	Y	Y	Correct	1894	N	N	Correct
769	N	N	Correct	1895	N	N	Correct
770	Y	Y	Correct	1896	Y	Y	Correct
771	Y	Y	Correct	1897	Y	Y	Correct
772	Y	Y	Correct	1898	Y	Y	Correct
773	Y	Y	Correct	1899	Y	Y	Correct
774	Y	Y	Correct	1900	Y	Y	Correct

775	N	N	Correct	1901	Y	Y	Correct
776	N	N	Correct	1902	Y	Y	Correct
777	N	N	Correct	1903	Y	Y	Correct
778	N	N	Correct	1904	Y	Y	Correct
779	N	N	Correct	1905	Y	Y	Correct
780	N	N	Correct	1906	Y	Y	Correct
781	N	N	Correct	1907	Y	Y	Correct
782	Y	Y	Correct	1908	Y	Y	Correct
783	Y	Y	Correct	1909	Y	Y	Correct
784	Y	Y	Correct	1910	Y	Y	Correct
785	N	N	Correct	1911	Y	Y	Correct
786	N	N	Correct	1912	Y	Y	Correct
787	N	N	Correct	1913	N	N	Correct
788	N	N	Correct	1914	Y	Y	Correct
789	N	N	Correct	1915	Y	Y	Correct
790	N	N	Correct	1916	Y	Y	Correct
791	N	N	Correct	1917	N	N	Correct
792	N	N	Correct	1918	Y	Y	Correct
793	N	N	Correct	1919	N	N	Correct
794	N	N	Correct	1920	N	N	Correct
795	N	N	Correct	1921	N	N	Correct
796	N	N	Correct	1922	N	N	Correct
797	N	N	Correct	1923	N	N	Correct
798	N	N	Correct	1924	N	N	Correct
799	N	N	Correct	1925	N	N	Correct
800	N	N	Correct	1926	N	N	Correct
801	N	N	Correct	1927	N	N	Correct
802	N	N	Correct	1928	N	N	Correct
803	N	N	Correct	1929	N	N	Correct
804	N	N	Correct	1930	N	N	Correct
805	N	N	Correct	1931	N	N	Correct
806	N	N	Correct	1932	N	N	Correct
807	N	N	Correct	1933	N	N	Correct
808	N	N	Correct	1934	N	N	Correct
809	N	N	Correct	1935	N	N	Correct
810	N	N	Correct	1936	N	N	Correct
811	N	N	Correct	1937	N	N	Correct
812	N	N	Correct	1938	N	N	Correct
813	N	N	Correct	1939	N	N	Correct
814	N	N	Correct	1940	N	N	Correct
815	N	N	Correct	1941	N	N	Correct

816	N	N	Correct	1942	N	N	Correct
817	N	N	Correct	1943	N	N	Correct
818	N	N	Correct	1944	N	N	Correct
819	N	N	Correct	1945	N	N	Correct
820	N	N	Correct	1946	N	N	Correct
821	N	N	Correct	1947	N	N	Correct
822	N	N	Correct	1948	N	N	Correct
823	N	N	Correct	1949	N	N	Correct
824	N	N	Correct	1950	N	N	Correct
825	N	N	Correct	1951	N	N	Correct
826	N	N	Correct	1952	N	N	Correct
827	N	N	Correct	1953	N	N	Correct
828	N	N	Correct	1954	N	N	Correct
829	N	N	Correct	1955	N	N	Correct
830	N	N	Correct	1956	N	N	Correct
831	N	N	Correct	1957	N	N	Correct
832	N	N	Correct	1958	Y	Y	Correct
833	N	N	Correct	1959	Y	Y	Correct
834	Y	Y	Correct	1960	Y	Y	Correct
835	Y	Y	Correct	1961	Y	Y	Correct
836	Y	Y	Correct	1962	N	N	Correct
837	Y	Y	Correct	1963	N	N	Correct
838	Y	Y	Correct	1964	N	N	Correct
839	Y	Y	Correct	1965	N	N	Correct
840	Y	Y	Correct	1966	N	N	Correct
841	Y	Y	Correct	1967	N	N	Correct
842	Y	Y	Correct	1968	N	N	Correct
843	N	N	Correct	1969	N	N	Correct
844	Y	Y	Correct	1970	N	N	Correct
845	Y	Y	Correct	1971	N	N	Correct
846	N	N	Correct	1972	N	N	Correct
847	N	N	Correct	1973	Y	Y	Correct
848	N	N	Correct	1974	Y	Y	Correct
849	N	N	Correct	1975	N	N	Correct
850	N	N	Correct	1976	N	N	Correct
851	N	N	Correct	1977	N	N	Correct
852	N	N	Correct	1978	N	N	Correct
853	N	N	Correct	1979	N	N	Correct
854	N	N	Correct	1980	N	N	Correct
855	N	N	Correct	1981	N	N	Correct
856	N	N	Correct	1982	N	N	Correct

857	N	N	Correct	1983	Y	Y	Correct
858	N	N	Correct	1984	Y	Y	Correct
859	N	N	Correct	1985	Y	Y	Correct
860	N	N	Correct	1986	Y	Y	Correct
861	N	N	Correct	1987	Y	Y	Correct
862	N	N	Correct	1988	Y	Y	Correct
863	N	N	Correct	1989	Y	Y	Correct
864	N	N	Correct	1990	Y	Y	Correct
865	N	N	Correct	1991	N	N	Correct
866	N	N	Correct	1992	N	N	Correct
867	N	N	Correct	1993	N	N	Correct
868	N	N	Correct	1994	N	N	Correct
869	N	N	Correct	1995	N	N	Correct
870	N	N	Correct	1996	N	N	Correct
871	N	N	Correct	1997	N	N	Correct
872	N	N	Correct	1998	N	N	Correct
873	N	N	Correct	1999	N	N	Correct
874	N	N	Correct	2000	N	N	Correct
875	N	N	Correct	2001	N	N	Correct
876	N	N	Correct	2002	N	N	Correct
877	N	N	Correct	2003	N	N	Correct
878	N	N	Correct	2004	N	N	Correct
879	N	N	Correct	2005	N	N	Correct
880	N	N	Correct	2006	N	N	Correct
881	N	N	Correct	2007	N	N	Correct
882	N	N	Correct	2008	N	N	Correct
883	N	N	Correct	2009	N	N	Correct
884	N	N	Correct	2010	N	N	Correct
885	N	N	Correct	2011	N	N	Correct
886	N	N	Correct	2012	N	N	Correct
887	N	N	Correct	2013	N	N	Correct
888	N	N	Correct	2014	N	N	Correct
889	N	N	Correct	2015	N	N	Correct
890	N	N	Correct	2016	N	N	Correct
891	N	N	Correct	2017	N	N	Correct
892	N	N	Correct	2018	N	N	Correct
893	N	N	Correct	2019	N	N	Correct
894	N	N	Correct	2020	N	N	Correct
895	N	N	Correct	2021	N	N	Correct
896	N	N	Correct	2022	N	N	Correct
897	N	N	Correct	2023	N	N	Correct

898	N	N	Correct	2024	N	N	Correct
899	N	N	Correct	2025	N	N	Correct
900	N	N	Correct	2026	N	N	Correct
901	N	N	Correct	2027	N	N	Correct
902	N	N	Correct	2028	N	N	Correct
903	N	N	Correct	2029	Y	Y	Correct
904	N	N	Correct	2030	Y	Y	Correct
905	N	N	Correct	2031	Y	Y	Correct
906	N	N	Correct	2032	Y	Y	Correct
907	N	N	Correct	2033	Y	Y	Correct
908	N	N	Correct	2034	Y	Y	Correct
909	N	N	Correct	2035	Y	Y	Correct
910	N	N	Correct	2036	Y	Y	Correct
911	N	N	Correct	2037	Y	Y	Correct
912	N	N	Correct	2038	Y	Y	Correct
913	N	N	Correct	2039	N	N	Correct
914	N	N	Correct	2040	Y	Y	Correct
915	N	N	Correct	2041	Y	Y	Correct
916	N	N	Correct	2042	Y	Y	Correct
917	N	N	Correct	2043	N	N	Correct
918	N	N	Correct	2044	N	N	Correct
919	N	N	Correct	2045	N	N	Correct
920	N	N	Correct	2046	N	N	Correct
921	N	N	Correct	2047	N	N	Correct
922	N	N	Correct	2048	N	N	Correct
923	N	N	Correct	2049	N	N	Correct
924	N	N	Correct	2050	N	N	Correct
925	N	N	Correct	2051	N	N	Correct
926	N	N	Correct	2052	N	N	Correct
927	N	N	Correct	2053	N	N	Correct
928	N	N	Correct	2054	N	N	Correct
929	N	N	Correct	2055	N	N	Correct
930	N	N	Correct	2056	N	N	Correct
931	N	N	Correct	2057	N	N	Correct
932	N	N	Correct	2058	N	N	Correct
933	N	N	Correct	2059	N	N	Correct
934	N	N	Correct	2060	N	N	Correct
935	N	N	Correct	2061	N	N	Correct
936	N	N	Correct	2062	N	N	Correct
937	N	N	Correct	2063	N	N	Correct
938	N	N	Correct	2064	N	N	Correct

939	N	N	Correct	2065	N	N	Correct
940	N	N	Correct	2066	N	N	Correct
941	N	N	Correct	2067	N	N	Correct
942	N	N	Correct	2068	N	N	Correct
943	N	N	Correct	2069	N	N	Correct
944	N	N	Correct	2070	N	N	Correct
945	N	N	Correct	2071	N	N	Correct
946	N	N	Correct	2072	N	N	Correct
947	N	N	Correct	2073	N	N	Correct
948	Y	Y	Correct	2074	N	N	Correct
949	Y	Y	Correct	2075	N	N	Correct
950	Y	Y	Correct	2076	N	N	Correct
951	Y	Y	Correct	2077	N	N	Correct
952	Y	Y	Correct	2078	N	N	Correct
953	Y	Y	Correct	2079	N	N	Correct
954	Y	Y	Correct	2080	N	N	Correct
955	Y	Y	Correct	2081	N	N	Correct
956	Y	Y	Correct	2082	N	N	Correct
957	Y	Y	Correct	2083	N	N	Correct
958	Y	Y	Correct	2084	N	N	Correct
959	Y	Y	Correct	2085	N	N	Correct
960	Y	Y	Correct	2086	N	N	Correct
961	Y	Y	Correct	2087	N	N	Correct
962	Y	Y	Correct	2088	N	N	Correct
963	Y	Y	Correct	2089	N	N	Correct
964	Y	Y	Correct	2090	N	N	Correct
965	Y	Y	Correct	2091	N	N	Correct
966	N	N	Correct	2092	N	N	Correct
967	Y	Y	Correct	2093	N	N	Correct
968	N	N	Correct	2094	N	N	Correct
969	N	N	Correct	2095	N	N	Correct
970	N	N	Correct	2096	N	N	Correct
971	N	N	Correct	2097	N	N	Correct
972	N	N	Correct	2098	N	N	Correct
973	N	N	Correct	2099	Y	Y	Correct
974	N	N	Correct	2100	N	N	Correct
975	N	N	Correct	2101	N	N	Correct
976	N	N	Correct	2102	Y	Y	Correct
977	N	N	Correct	2103	Y	Y	Correct
978	N	N	Correct	2104	Y	Y	Correct
979	N	N	Correct	2105	Y	Y	Correct

980	N	N	Correct	2106	Y	Y	Correct
981	N	N	Correct	2107	Y	Y	Correct
982	N	N	Correct	2108	Y	Y	Correct
983	N	N	Correct	2109	Y	Y	Correct
984	N	N	Correct	2110	Y	Y	Correct
985	N	N	Correct	2111	Y	Y	Correct
986	N	N	Correct	2112	N	N	Correct
987	N	N	Correct	2113	N	N	Correct
988	N	N	Correct	2114	N	N	Correct
989	N	N	Correct	2115	N	N	Correct
990	N	N	Correct	2116	N	N	Correct
991	N	N	Correct	2117	N	N	Correct
992	N	N	Correct	2118	N	N	Correct
993	N	N	Correct	2119	N	N	Correct
994	N	N	Correct	2120	Y	Y	Correct
995	N	N	Correct	2121	Y	Y	Correct
996	N	N	Correct	2122	Y	Y	Correct
997	N	N	Correct	2123	Y	Y	Correct
998	N	N	Correct	2124	Y	Y	Correct
999	N	N	Correct	2125	Y	Y	Correct
1000	N	N	Correct	2126	Y	Y	Correct
1001	N	N	Correct	2127	N	N	Correct
1002	N	N	Correct	2128	N	N	Correct
1003	Y	Y	Correct	2129	N	N	Correct
1004	Y	Y	Correct	2130	N	N	Correct
1005	Y	Y	Correct	2131	N	N	Correct
1006	Y	Y	Correct	2132	N	Y	Mismatch
1007	Y	Y	Correct	2133	Y	Y	Correct
1008	Y	Y	Correct	2134	Y	Y	Correct
1009	Y	Y	Correct	2135	Y	Y	Correct
1010	Y	Y	Correct	2136	Y	Y	Correct
1011	Y	Y	Correct	2137	N	N	Correct
1012	Y	Y	Correct	2138	N	N	Correct
1013	Y	Y	Correct	2139	N	N	Correct
1014	N	N	Correct	2140	N	N	Correct
1015	N	N	Correct	2141	N	N	Correct
1016	N	N	Correct	2142	N	N	Correct
1017	N	N	Correct	2143	N	N	Correct
1018	Y	Y	Correct	2144	N	N	Correct
1019	Y	Y	Correct	2145	N	N	Correct
1020	Y	Y	Correct	2146	N	N	Correct

1021	Y	Y	Correct	2147	N	N	Correct
1022	N	N	Correct	2148	N	N	Correct
1023	N	N	Correct	2149	N	N	Correct
1024	N	N	Correct	2150	N	N	Correct
1025	N	N	Correct	2151	N	N	Correct
1026	N	N	Correct	2152	N	N	Correct
1027	N	N	Correct	2153	N	N	Correct
1028	N	N	Correct	2154	N	N	Correct
1029	N	N	Correct	2155	N	N	Correct
1030	N	N	Correct	2156	N	N	Correct
1031	N	N	Correct	2157	N	N	Correct
1032	N	N	Correct	2158	N	N	Correct
1033	N	N	Correct	2159	N	N	Correct
1034	N	N	Correct	2160	N	N	Correct
1035	N	N	Correct	2161	N	N	Correct
1036	N	N	Correct	2162	N	N	Correct
1037	N	N	Correct	2163	N	N	Correct
1038	N	N	Correct	2164	N	N	Correct
1039	N	N	Correct	2165	N	N	Correct
1040	N	N	Correct	2166	N	N	Correct
1041	N	N	Correct	2167	N	N	Correct
1042	N	N	Correct	2168	N	N	Correct
1043	N	N	Correct	2169	N	N	Correct
1044	N	N	Correct	2170	Y	Y	Correct
1045	N	N	Correct	2171	Y	Y	Correct
1046	N	N	Correct	2172	Y	Y	Correct
1047	N	N	Correct	2173	Y	Y	Correct
1048	N	N	Correct	2174	Y	Y	Correct
1049	N	N	Correct	2175	Y	Y	Correct
1050	N	N	Correct	2176	Y	Y	Correct
1051	N	N	Correct	2177	Y	Y	Correct
1052	N	N	Correct	2178	Y	Y	Correct
1053	N	N	Correct	2179	Y	Y	Correct
1054	N	N	Correct	2180	Y	Y	Correct
1055	N	N	Correct	2181	Y	Y	Correct
1056	N	N	Correct	2182	Y	Y	Correct
1057	N	N	Correct	2183	Y	Y	Correct
1058	N	N	Correct	2184	N	N	Correct
1059	N	N	Correct	2185	Y	Y	Correct
1060	N	N	Correct	2186	Y	Y	Correct
1061	Y	Y	Correct	2187	N	N	Correct

1062	Y	Y	Correct	2188	N	N	Correct
1063	Y	Y	Correct	2189	N	N	Correct
1064	Y	Y	Correct	2190	N	N	Correct
1065	Y	Y	Correct	2191	N	N	Correct
1066	N	N	Correct	2192	N	N	Correct
1067	Y	Y	Correct	2193	N	N	Correct
1068	N	N	Correct	2194	N	N	Correct
1069	N	N	Correct	2195	N	N	Correct
1070	N	N	Correct	2196	N	N	Correct
1071	N	N	Correct	2197	N	N	Correct
1072	N	N	Correct	2198	N	N	Correct
1073	N	N	Correct	2199	N	N	Correct
1074	N	N	Correct	2200	N	N	Correct
1075	Y	Y	Correct	2201	N	N	Correct
1076	Y	Y	Correct	2202	N	N	Correct
1077	N	N	Correct	2203	N	N	Correct
1078	N	N	Correct	2204	N	N	Correct
1079	Y	Y	Correct	2205	N	N	Correct
1080	Y	Y	Correct	2206	N	N	Correct
1081	Y	Y	Correct	2207	N	N	Correct
1082	N	N	Correct	2208	N	N	Correct
1083	N	N	Correct	2209	N	N	Correct
1084	N	N	Correct	2210	N	N	Correct
1085	N	N	Correct	2211	N	N	Correct
1086	N	N	Correct	2212	N	N	Correct
1087	N	N	Correct	2213	N	N	Correct
1088	N	N	Correct	2214	N	N	Correct
1089	Y	Y	Correct	2215	N	N	Correct
1090	Y	Y	Correct	2216	N	N	Correct
1091	Y	Y	Correct	2217	N	N	Correct
1092	Y	Y	Correct	2218	N	N	Correct
1093	Y	Y	Correct	2219	N	N	Correct
1094	Y	Y	Correct	2220	N	N	Correct
1095	Y	Y	Correct	2221	N	N	Correct
1096	Y	Y	Correct	2222	N	N	Correct
1097	N	N	Correct	2223	N	N	Correct
1098	N	N	Correct	2224	N	N	Correct
1099	N	N	Correct	2225	N	N	Correct
1100	N	N	Correct	2226	N	N	Correct
1101	N	N	Correct	2227	N	N	Correct
1102	N	N	Correct	2228	N	N	Correct

1103	N	N	Correct	2229	N	N	Correct
1104	N	N	Correct	2230	N	N	Correct
1105	N	N	Correct	2231	N	N	Correct
1106	N	N	Correct	2232	N	N	Correct
1107	N	N	Correct	2233	N	N	Correct
1108	N	N	Correct	2234	N	N	Correct
1109	N	N	Correct	2235	N	N	Correct
1110	N	N	Correct	2236	N	N	Correct
1111	N	N	Correct	2237	N	N	Correct
1112	N	N	Correct	2238	N	N	Correct
1113	N	N	Correct	2239	N	N	Correct
1114	N	N	Correct	2240	N	N	Correct
1115	N	N	Correct	2241	N	N	Correct
1116	N	N	Correct	2242	N	N	Correct
1117	N	N	Correct	2243	N	N	Correct
1118	N	N	Correct	2244	N	N	Correct
1119	N	N	Correct	2245	N	N	Correct
1120	N	N	Correct	2246	N	N	Correct
1121	N	N	Correct	2247	N	N	Correct
1122	N	N	Correct	2248	N	N	Correct
1123	N	N	Correct	2249	N	N	Correct
1124	N	N	Correct	2250	N	N	Correct
1125	N	N	Correct	2251	Y	Y	Correct
1126	N	N	Correct				

Supplementary Table S3. Prediction results of the external validation phase (Phase 2) of the Random Forest model for multidrug resistance in *Campylobacter* isolates from swine (Actual vs. Predicted)

No.	Predicted	Actual	Prediction result	No.	Predicted	Actual	Prediction result
1	N	N	Correct	303	N	N	Correct
2	N	N	Correct	304	N	N	Correct
3	N	N	Correct	305	N	N	Correct
4	N	N	Correct	306	N	N	Correct
5	N	N	Correct	307	N	N	Correct
6	N	N	Correct	308	Y	Y	Correct
7	N	N	Correct	309	Y	Y	Correct
8	N	N	Correct	310	Y	Y	Correct
9	N	N	Correct	311	Y	Y	Correct
10	N	N	Correct	312	Y	Y	Correct
11	N	N	Correct	313	Y	Y	Correct
12	N	N	Correct	314	Y	Y	Correct
13	N	N	Correct	315	Y	Y	Correct
14	N	N	Correct	316	Y	Y	Correct
15	N	N	Correct	317	Y	Y	Correct
16	N	N	Correct	318	Y	Y	Correct
17	N	N	Correct	319	Y	Y	Correct
18	N	N	Correct	320	Y	Y	Correct
19	N	N	Correct	321	Y	Y	Correct
20	N	N	Correct	322	Y	Y	Correct
21	N	N	Correct	323	N	N	Correct
22	N	N	Correct	324	Y	Y	Correct
23	N	N	Correct	325	Y	Y	Correct
24	N	N	Correct	326	N	N	Correct
25	N	N	Correct	327	Y	Y	Correct
26	N	N	Correct	328	Y	Y	Correct
27	N	N	Correct	329	N	N	Correct
28	N	N	Correct	330	N	N	Correct
29	N	N	Correct	331	N	Y	Mismatch
30	N	N	Correct	332	N	N	Correct
31	N	Y	Mismatch	333	N	N	Correct
32	N	N	Correct	334	N	N	Correct
33	N	N	Correct	335	N	N	Correct
34	N	N	Correct	336	N	N	Correct
35	N	N	Correct	337	N	N	Correct
36	N	N	Correct	338	N	N	Correct

37	N	N	Correct	339	N	N	Correct
38	N	N	Correct	340	N	N	Correct
39	N	N	Correct	341	N	N	Correct
40	N	N	Correct	342	N	N	Correct
41	N	N	Correct	343	N	N	Correct
42	N	N	Correct	344	N	N	Correct
43	N	N	Correct	345	N	N	Correct
44	N	N	Correct	346	N	N	Correct
45	N	N	Correct	347	N	N	Correct
46	N	N	Correct	348	N	N	Correct
47	N	N	Correct	349	N	N	Correct
48	N	N	Correct	350	N	N	Correct
49	N	N	Correct	351	N	N	Correct
50	N	N	Correct	352	N	N	Correct
51	N	N	Correct	353	N	N	Correct
52	N	N	Correct	354	N	N	Correct
53	N	N	Correct	355	N	N	Correct
54	N	N	Correct	356	N	N	Correct
55	N	N	Correct	357	N	N	Correct
56	N	N	Correct	358	N	N	Correct
57	N	N	Correct	359	N	N	Correct
58	N	N	Correct	360	N	N	Correct
59	N	N	Correct	361	N	N	Correct
60	N	N	Correct	362	N	N	Correct
61	N	N	Correct	363	N	N	Correct
62	N	N	Correct	364	N	N	Correct
63	N	Y	Mismatch	365	N	N	Correct
64	N	N	Correct	366	N	N	Correct
65	N	N	Correct	367	N	N	Correct
66	N	N	Correct	368	N	N	Correct
67	N	N	Correct	369	N	N	Correct
68	N	N	Correct	370	N	N	Correct
69	Y	Y	Correct	371	N	N	Correct
70	Y	Y	Correct	372	N	N	Correct
71	Y	Y	Correct	373	N	N	Correct
72	Y	Y	Correct	374	N	N	Correct
73	Y	Y	Correct	375	N	N	Correct
74	Y	Y	Correct	376	N	N	Correct
75	Y	Y	Correct	377	N	N	Correct
76	Y	Y	Correct	378	N	N	Correct
77	Y	Y	Correct	379	N	N	Correct

78	Y	Y	Correct	380	N	N	Correct
79	Y	Y	Correct	381	N	N	Correct
80	Y	Y	Correct	382	N	N	Correct
81	Y	Y	Correct	383	N	N	Correct
82	Y	Y	Correct	384	N	N	Correct
83	Y	Y	Correct	385	N	N	Correct
84	Y	Y	Correct	386	N	N	Correct
85	Y	Y	Correct	387	N	N	Correct
86	Y	Y	Correct	388	N	N	Correct
87	Y	Y	Correct	389	N	N	Correct
88	Y	Y	Correct	390	N	N	Correct
89	Y	Y	Correct	391	N	N	Correct
90	Y	Y	Correct	392	N	N	Correct
91	Y	Y	Correct	393	N	N	Correct
92	Y	Y	Correct	394	N	N	Correct
93	Y	Y	Correct	395	N	N	Correct
94	Y	Y	Correct	396	N	N	Correct
95	Y	Y	Correct	397	N	N	Correct
96	Y	Y	Correct	398	N	N	Correct
97	Y	Y	Correct	399	N	N	Correct
98	Y	Y	Correct	400	N	N	Correct
99	Y	Y	Correct	401	N	N	Correct
100	Y	Y	Correct	402	N	N	Correct
101	Y	Y	Correct	403	N	N	Correct
102	Y	Y	Correct	404	N	N	Correct
103	Y	Y	Correct	405	N	N	Correct
104	Y	Y	Correct	406	N	N	Correct
105	N	N	Correct	407	N	N	Correct
106	N	N	Correct	408	N	N	Correct
107	N	N	Correct	409	N	N	Correct
108	N	N	Correct	410	N	N	Correct
109	N	N	Correct	411	N	N	Correct
110	N	N	Correct	412	N	N	Correct
111	N	N	Correct	413	N	N	Correct
112	N	N	Correct	414	N	N	Correct
113	N	N	Correct	415	N	N	Correct
114	N	N	Correct	416	N	N	Correct
115	N	N	Correct	417	Y	Y	Correct
116	N	N	Correct	418	N	N	Correct
117	N	N	Correct	419	N	N	Correct
118	N	N	Correct	420	N	N	Correct

119	N	N	Correct	421	N	N	Correct
120	N	N	Correct	422	N	N	Correct
121	N	N	Correct	423	Y	Y	Correct
122	N	Y	Mismatch	424	N	N	Correct
123	N	N	Correct	425	N	N	Correct
124	N	N	Correct	426	N	N	Correct
125	N	N	Correct	427	N	N	Correct
126	N	N	Correct	428	N	N	Correct
127	N	N	Correct	429	N	N	Correct
128	Y	Y	Correct	430	N	N	Correct
129	Y	Y	Correct	431	N	N	Correct
130	Y	Y	Correct	432	N	N	Correct
131	Y	Y	Correct	433	N	N	Correct
132	Y	Y	Correct	434	N	N	Correct
133	Y	Y	Correct	435	N	N	Correct
134	Y	Y	Correct	436	N	N	Correct
135	Y	Y	Correct	437	N	N	Correct
136	Y	Y	Correct	438	N	N	Correct
137	Y	Y	Correct	439	N	N	Correct
138	Y	Y	Correct	440	N	N	Correct
139	Y	Y	Correct	441	Y	Y	Correct
140	Y	Y	Correct	442	N	N	Correct
141	Y	Y	Correct	443	N	N	Correct
142	Y	Y	Correct	444	Y	Y	Correct
143	N	N	Correct	445	N	N	Correct
144	N	N	Correct	446	N	N	Correct
145	N	N	Correct	447	N	N	Correct
146	N	N	Correct	448	Y	Y	Correct
147	N	N	Correct	449	Y	Y	Correct
148	N	N	Correct	450	N	N	Correct
149	N	N	Correct	451	N	N	Correct
150	N	N	Correct	452	Y	Y	Correct
151	N	N	Correct	453	N	N	Correct
152	N	N	Correct	454	Y	Y	Correct
153	N	N	Correct	455	N	N	Correct
154	N	N	Correct	456	N	N	Correct
155	N	N	Correct	457	N	N	Correct
156	N	N	Correct	458	N	N	Correct
157	N	N	Correct	459	Y	Y	Correct
158	N	N	Correct	460	N	N	Correct
159	N	N	Correct	461	N	N	Correct

160	N	N	Correct	462	N	N	Correct
161	N	N	Correct	463	Y	Y	Correct
162	N	N	Correct	464	N	N	Correct
163	N	N	Correct	465	Y	Y	Correct
164	N	N	Correct	466	N	N	Correct
165	N	N	Correct	467	Y	Y	Correct
166	N	N	Correct	468	N	N	Correct
167	N	N	Correct	469	Y	Y	Correct
168	N	N	Correct	470	Y	Y	Correct
169	N	N	Correct	471	N	N	Correct
170	N	N	Correct	472	N	N	Correct
171	N	N	Correct	473	N	N	Correct
172	N	N	Correct	474	N	N	Correct
173	N	N	Correct	475	N	N	Correct
174	N	N	Correct	476	N	N	Correct
175	N	N	Correct	477	N	N	Correct
176	N	N	Correct	478	N	N	Correct
177	N	N	Correct	479	N	N	Correct
178	N	N	Correct	480	N	N	Correct
179	N	N	Correct	481	N	N	Correct
180	N	N	Correct	482	N	N	Correct
181	N	N	Correct	483	N	N	Correct
182	N	N	Correct	484	N	N	Correct
183	N	N	Correct	485	N	N	Correct
184	N	N	Correct	486	N	N	Correct
185	N	N	Correct	487	Y	Y	Correct
186	N	N	Correct	488	N	N	Correct
187	N	N	Correct	489	N	N	Correct
188	N	N	Correct	490	N	N	Correct
189	N	N	Correct	491	N	Y	Mismatch
190	N	N	Correct	492	Y	Y	Correct
191	N	N	Correct	493	N	N	Correct
192	N	N	Correct	494	Y	Y	Correct
193	N	N	Correct	495	N	N	Correct
194	N	N	Correct	496	Y	Y	Correct
195	N	N	Correct	497	Y	Y	Correct
196	N	N	Correct	498	N	N	Correct
197	N	N	Correct	499	N	N	Correct
198	N	N	Correct	500	N	N	Correct
199	N	N	Correct	501	N	N	Correct
200	N	N	Correct	502	N	N	Correct

201	N	N	Correct	503	N	N	Correct
202	N	N	Correct	504	N	N	Correct
203	N	N	Correct	505	N	N	Correct
204	N	N	Correct	506	N	N	Correct
205	N	N	Correct	507	N	N	Correct
206	N	N	Correct	508	N	N	Correct
207	N	N	Correct	509	N	N	Correct
208	N	N	Correct	510	Y	Y	Correct
209	N	N	Correct	511	N	N	Correct
210	N	N	Correct	512	Y	Y	Correct
211	N	N	Correct	513	N	N	Correct
212	N	N	Correct	514	N	N	Correct
213	N	N	Correct	515	N	N	Correct
214	N	N	Correct	516	N	N	Correct
215	N	N	Correct	517	N	N	Correct
216	N	N	Correct	518	N	N	Correct
217	N	N	Correct	519	N	N	Correct
218	N	N	Correct	520	N	N	Correct
219	N	N	Correct	521	Y	Y	Correct
220	N	Y	Mismatch	522	Y	Y	Correct
221	N	N	Correct	523	Y	Y	Correct
222	N	N	Correct	524	N	N	Correct
223	N	N	Correct	525	Y	Y	Correct
224	N	N	Correct	526	N	N	Correct
225	N	N	Correct	527	N	N	Correct
226	N	N	Correct	528	N	N	Correct
227	N	N	Correct	529	N	N	Correct
228	N	N	Correct	530	N	N	Correct
229	N	N	Correct	531	N	N	Correct
230	N	N	Correct	532	N	N	Correct
231	N	N	Correct	533	N	N	Correct
232	N	N	Correct	534	N	N	Correct
233	N	N	Correct	535	N	N	Correct
234	N	N	Correct	536	N	N	Correct
235	N	N	Correct	537	N	N	Correct
236	N	N	Correct	538	N	N	Correct
237	N	N	Correct	539	N	N	Correct
238	N	N	Correct	540	N	N	Correct
239	N	N	Correct	541	Y	Y	Correct
240	N	N	Correct	542	N	Y	Mismatch
241	N	N	Correct	543	N	N	Correct

242	N	N	Correct	544	N	N	Correct
243	N	N	Correct	545	N	N	Correct
244	N	N	Correct	546	N	N	Correct
245	N	N	Correct	547	Y	Y	Correct
246	N	N	Correct	548	N	N	Correct
247	N	N	Correct	549	N	N	Correct
248	N	N	Correct	550	Y	Y	Correct
249	N	N	Correct	551	N	N	Correct
250	N	N	Correct	552	N	N	Correct
251	N	N	Correct	553	Y	Y	Correct
252	N	N	Correct	554	N	N	Correct
253	N	N	Correct	555	N	N	Correct
254	N	N	Correct	556	Y	Y	Correct
255	N	N	Correct	557	Y	Y	Correct
256	N	N	Correct	558	N	N	Correct
257	N	N	Correct	559	N	N	Correct
258	N	N	Correct	560	N	N	Correct
259	Y	Y	Correct	561	N	N	Correct
260	Y	Y	Correct	562	N	N	Correct
261	Y	Y	Correct	563	N	N	Correct
262	Y	Y	Correct	564	N	N	Correct
263	Y	Y	Correct	565	N	N	Correct
264	N	Y	Mismatch	566	N	N	Correct
265	Y	Y	Correct	567	Y	Y	Correct
266	Y	Y	Correct	568	N	N	Correct
267	Y	Y	Correct	569	N	N	Correct
268	N	N	Correct	570	N	N	Correct
269	Y	Y	Correct	571	N	N	Correct
270	Y	Y	Correct	572	N	N	Correct
271	N	N	Correct	573	Y	Y	Correct
272	Y	Y	Correct	574	N	N	Correct
273	Y	Y	Correct	575	N	N	Correct
274	Y	Y	Correct	576	N	N	Correct
275	Y	Y	Correct	577	N	N	Correct
276	Y	Y	Correct	578	N	N	Correct
277	Y	Y	Correct	579	N	N	Correct
278	Y	Y	Correct	580	N	N	Correct
279	Y	Y	Correct	581	N	N	Correct
280	Y	Y	Correct	582	N	N	Correct
281	N	N	Correct	583	N	N	Correct
282	N	N	Correct	584	N	N	Correct

283	N	N	Correct	585	N	N	Correct
284	N	N	Correct	586	Y	Y	Correct
285	N	N	Correct	587	N	N	Correct
286	N	N	Correct	588	N	N	Correct
287	N	N	Correct	589	Y	Y	Correct
288	N	N	Correct	590	Y	Y	Correct
289	N	N	Correct	591	N	N	Correct
290	N	N	Correct	592	N	N	Correct
291	N	N	Correct	593	N	N	Correct
292	N	N	Correct	594	N	N	Correct
293	N	N	Correct	595	N	N	Correct
294	N	N	Correct	596	N	N	Correct
295	N	N	Correct	597	Y	Y	Correct
296	N	Y	Mismatch	598	N	N	Correct
297	N	N	Correct	599	N	N	Correct
298	N	N	Correct	600	Y	Y	Correct
299	N	N	Correct	601	Y	Y	Correct
300	N	N	Correct	602	N	N	Correct
301	N	N	Correct	603	N	N	Correct
302	N	N	Correct				
